# One to All: Toward a Unified Model for Counting Cereal Crop Heads Based on Few-Shot Learning

**DOI:** 10.34133/plantphenomics.0271

**Published:** 2024-11-28

**Authors:** Qiang Wang, Xijian Fan, Ziqing Zhuang, Tardi Tjahjadi, Shichao Jin, Honghua Huan, Qiaolin Ye

**Affiliations:** ^1^ Nanjing Forestry University, Nanjing 210037, China.; ^2^ University of Warwick, Coventry CV4 7AL, UK.; ^3^ Crop Phenomics Research Centre, Academy for Advanced Interdisciplinary Studies, Collaborative Innovation Centre for Modern Crop Production cosponsored by Province and Ministry, State Key Laboratory of Crop Genetics and Germplasm Enhancement, Nanjing Agricultural University, Nanjing 210095, China.; ^4^ Jiangsu Academy of Agricultural Sciences, Nanjing 210014, China.

## Abstract

Accurate counting of cereals crops, e.g., maize, rice, sorghum, and wheat, is crucial for estimating grain production and ensuring food security. However, existing methods for counting cereal crops focus predominantly on building models for specific crop head; thus, they lack generalizability to different crop varieties. This paper presents Counting Heads of Cereal Crops Net (CHCNet), which is a unified model designed for counting multiple cereal crop heads by few-shot learning, which effectively reduces labeling costs. Specifically, a refined vision encoder is developed to enhance feature embedding, where a foundation model, namely, the segment anything model (SAM), is employed to emphasize the marked crop heads while mitigating complex background effects. Furthermore, a multiscale feature interaction module is proposed for integrating a similarity metric to facilitate automatic learning of crop-specific features across varying scales, which enhances the ability to describe crop heads of various sizes and shapes. The CHCNet model adopts a 2-stage training procedure. The initial stage focuses on latent feature mining to capture common feature representations of cereal crops. In the subsequent stage, inference is performed without additional training, by extracting domain-specific features of the target crop from selected exemplars to accomplish the counting task. In extensive experiments on 6 diverse crop datasets captured from ground cameras and drones, CHCNet substantially outperformed state-of-the-art counting methods in terms of cross-crop generalization ability, achieving mean absolute errors (MAEs) of 9.96 and 9.38 for maize, 13.94 for sorghum, 7.94 for rice, and 15.62 for mixed crops. A user-friendly interactive demo is available at http://cerealcropnet.com/, where researchers are invited to personally evaluate the proposed CHCNet. The source code for implementing CHCNet is available at https://github.com/Small-flyguy/CHCNet.

## Introduction

Accurate counting of cereal crops, e.g., maize, rice, sorghum, and wheat, is essential for estimating grain production and ensuring food security [1]. By precisely quantifying the number of crop heads, farmers and researchers can gain valuable insights into various crucial aspects of crop development and yield estimation. Moreover, crop head count serves as a fundamental parameter in breeding programs aimed at cultivating high-yielding and resilient crop varieties [2]. Thus, the pursuit of accurate and efficient crop head counting has always been a focal point of research in the agricultural domain.

Traditional cereal crop head counting methods fall into 2 categories: manual counting [3,4] and image processing-based techniques [5–7]. Manual counting, which depends on visual inspection, is labor intensive, time consuming, and prone to errors due to subjective human observation. Thus, it is unsuitable for large-scale agricultural contexts. Image processing techniques involve extracting handcrafted features, e.g., color, texture, and morphology, and converting counting into a classification problem via machine learning. However, the reliance of these methods on predefined features leads to inefficiencies when faced with nonuniform lighting conditions and backgrounds, thus making the methods unreliable in practical field settings.

In recent years, rapid advancements in deep learning have led to its widespread application in agricultural tasks, including crop counting [8–11]. Owing to the exceptional ability of convolutional neural networks (CNNs) to capture 2-dimensional (2D) spatial information, CNN-based counting methods outperform conventional image processing techniques. These methods predominantly encompass 3 approaches: segmentation, object detection, and density map-based regression. Segmentation-based counting leverages pixel-level network training to achieve precise separation between crops and background at the pixel level, facilitating accurate delineation of object boundaries [12–15]. However, this approach necessitates pixel-level annotations, requiring extensive time and effort owing to the need for detailed labeling. Object detection-based counting methods identify and classify objects by generating candidate regions and computing scores [4,16–19]; however, their performance diminishes in densely populated areas. Density map-based regression methods establish a direct correlation between local image features and object quantity, creating a mapping between the original image and a corresponding density map [20–25]. Density map-based methods excel in precisely locating individual crop heads within an image, especially in scenes with dense occlusions.

Owing to their promising counting performance, density map methods have gained prominence in counting various cereal crop heads identified by botanical names, e.g., maize tassels [9,26], rice panicles [27], sorghum panicles [28,29], and wheat spikes [30]. However, existing density map methods for counting cereal crops focus predominantly on building counting models for a specific type of crop head. Regrettably, a model trained on one crop fails to generalize to different crop types. One contributing factor to this constraint stems from the notable morphological variations among different cereal crops, although they are broadly categorized as crop plants. Thus, the development of head counting models for new crops necessitates the reannotation of samples and subsequent training of deep learning models, which entails substantial costs. To date, no model capable of counting multiple crop heads simultaneously has been developed. In addition, even between crop heads of the same type, the counting performance of established models may not transfer well due to variations in the data acquisition environment, such as shooting angle, distance, and time, which is a phenomenon known as domain shift [31]. Fine-tuning strategies in transfer learning can help alleviate this issue to some extent. For example, Liu et al. [32] proposed fine-tuning a wheat head counting model by training it on ground-based datasets and incorporating unmanned aerial vehicle (UAV)-based samples to facilitate model transfer effectively. Furthermore, the fine-tuning process requires annotated samples from the target dataset (domain) for training. Thus, many point annotations remain necessary for density map estimation methods. Considering the preceding discussion, is it possible to develop a unified counting model at a low annotation cost that is capable of handling various types of crop heads and diverse data acquisition environments, such as UAVs or ground-based cameras?

Humans can effectively generalize new concepts and identify objects of similar classes in query images, even when these objects differ in shape and are viewed under different illuminations, scales, and other factors. Motivated by the human capability of rapid concept generalization, few-shot counting (FSC) [33] was introduced to address the challenge of generalization. FSC can enumerate novel classes that are absent during the training phase by focusing on the user’s selection of random exemplars, solely counting the objects of corresponding classes in the query images. During training, only the base classes that are seen are utilized, with the inference conducted on the novel classes that are previously unseen, enabling the use of FSC methods, which have been recently introduced [34–36]. In this work, FSC techniques are employed to develop a unified label-efficient model for counting cereal crop heads, including maize tassels, rice panicles, sorghum panicles, and wheat spikes, in various data acquisition environments, namely, on-field ground-based cameras and close-range UAVs.

**Fig. 1.**
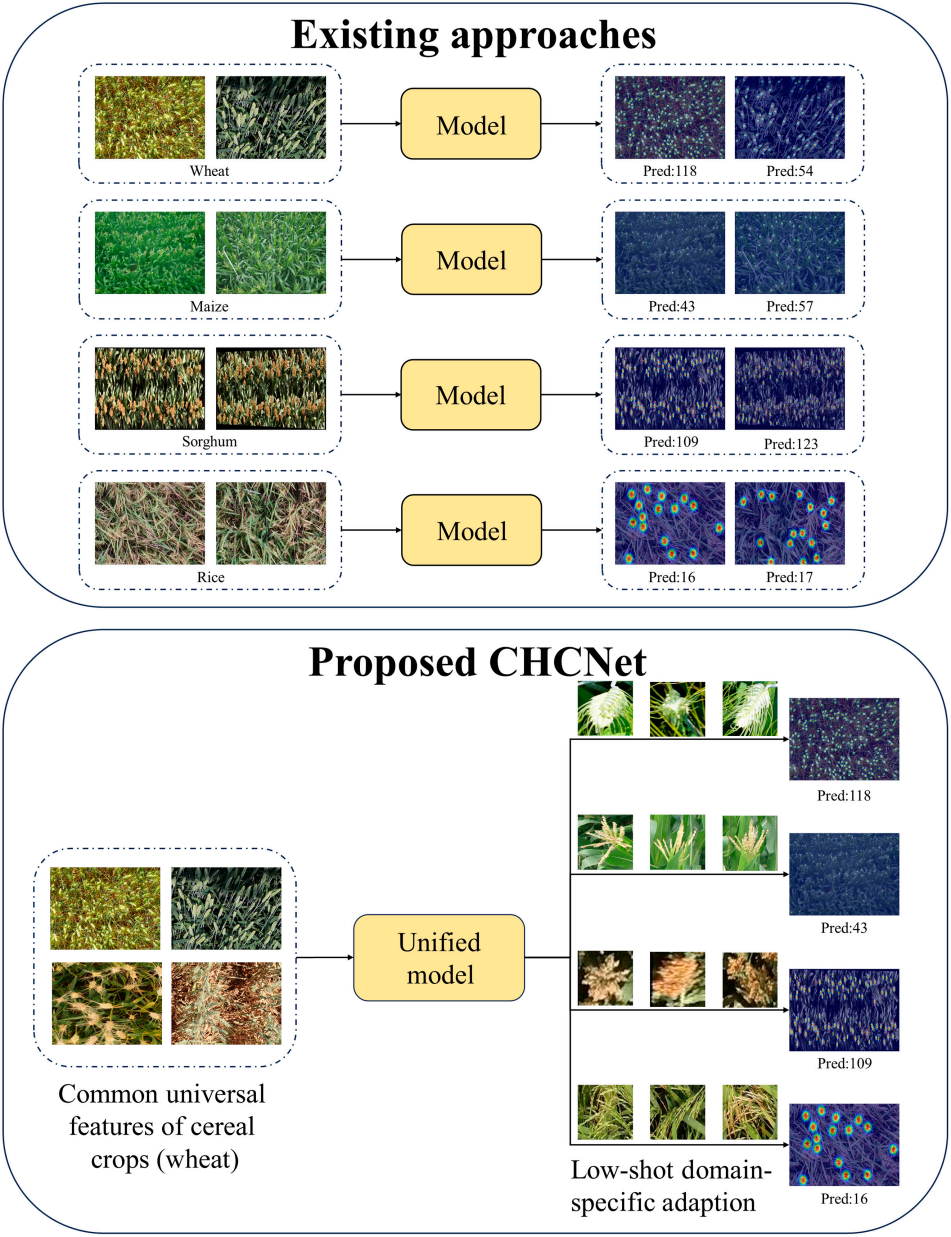
Comparative illustration of our method versus existing approaches. The proposed CHCNet is capable of acquiring the common features of cereal crops during the training phase and adapts to domain-specific features during inference without the need for additional training. This allows for a single training phase to suffice for adaptive counting across various types of cereal crops.

**Fig. 2.**
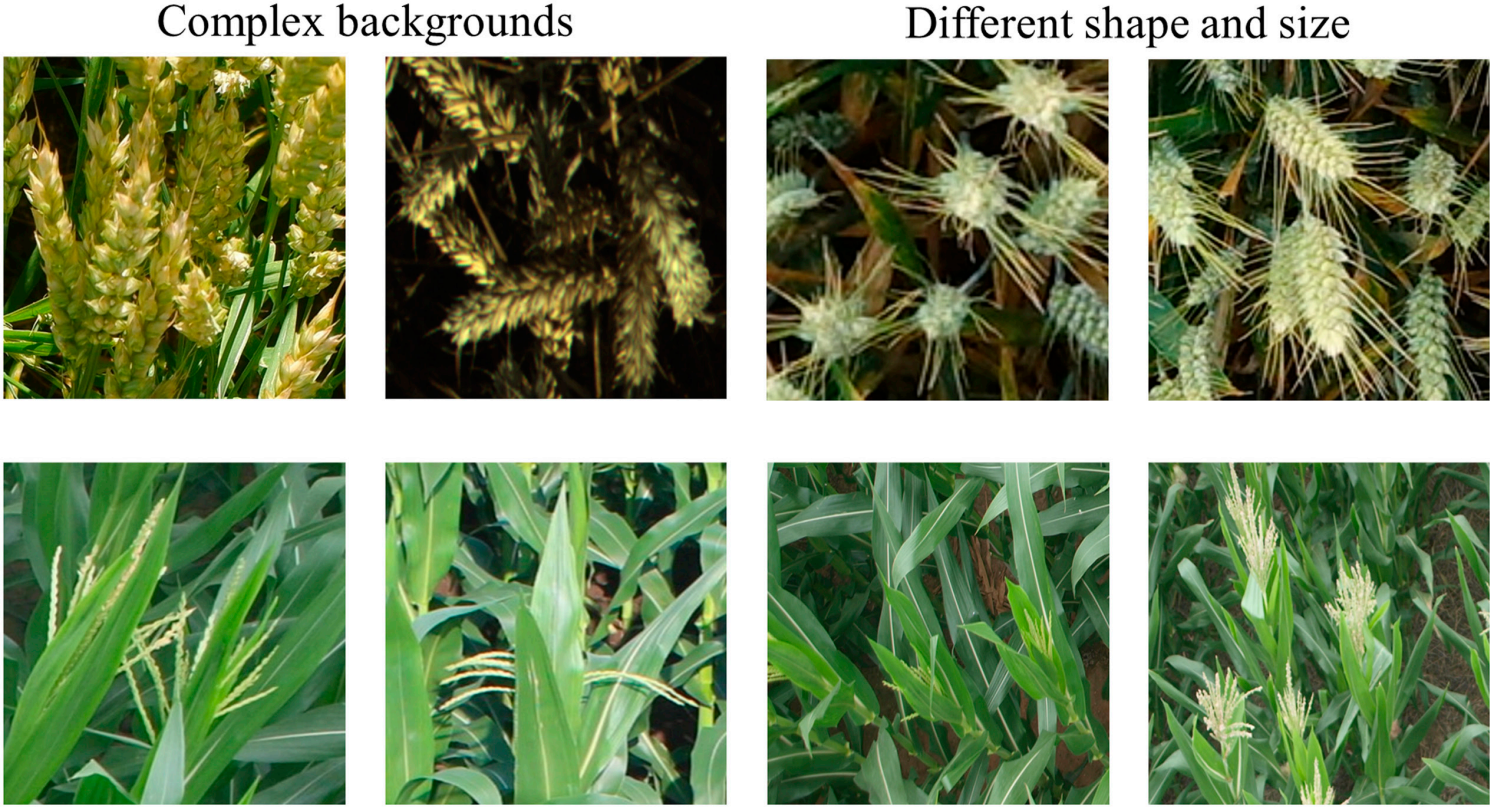
Illustrations of the challenges encountered in cereal crop counting scenarios, including complex backgrounds that introduce occlusions and the presence of objects at various scales within the images.

In the cereal crop head counting task, 2 pervasive challenges can significantly impact counting performance: the complexity of the background, which is characterized by leaf presence and overlapping instances, and variations in head size in images, which is attributed to camera shooting distance and the growth period of the crop, as illustrated in Fig. 1. To address interference due to the complex background, a refined vision encoder (RVE) module is specifically designed to extract the common characteristics of the crop-head object while filtering out irrelevant background elements. To address the variability in object size, a multiscale interaction module based on similarity learning has been devised to adaptively learn the inherent relationships between exemplars and image regions. This module streamlines the generation of similarity feature maps across various scales through adaptive acquisition of domain-specific knowledge. To achieve universal counting of cereal crops, a novel 2-stage scheme is proposed to establish a unified cereal crop counting method. In the first stage, the method engages in the latent feature mining through offline training to identify universal features that are common across cereal crops. The subsequent stage leverages a few-shot learning paradigm to extract domain-specific characteristics of the target scenes. A similarity comparison network is then utilized to generate a similarity feature map that embodies these domain-specific features. This map is subsequently decoded against the learned universal features of cereal crops for accurate counting. The novel aspects of our approach, in contrast to previous methods, are depicted in Fig. 2.

To summarize, the main contributions of this study are as follows:

• A unified 2-stage framework for counting cereal crop heads on the basis of few-shot learning is proposed, which significantly reduces the label cost for density map-based counting tasks. The proposed framework utilizes a novel strategy in the field of cereal crop head counting and offers marked advantages.

• An RVE is engineered to effectively enhance feature embedding, where the vision foundation model SAM (segment anything model) is employed to focus attention on the salient head target while reducing the effects of a complex background, e.g., leaves and stems.

• A multiscale feature interaction module that incorporates similarity learning is proposed, which enables the model to automatically learn domain-specific features and similarities between exemplars and image regions across different scales, thereby generating information-rich similarity feature maps.

• A new dataset of cereal crop heads is presented, which includes 4 types of cereal crops, namely, wheat, maize, sorghum, and rice, under different data acquisition environments. This dataset includes point annotations for each head and serves as a vital resource for assessing the efficacy of FSC techniques.

## Related Work

### Density map-based crop head counting

Few studies have focused on counting crop heads on the basis of density map estimation. Counting crop heads typically involves 4 main types of crops: maize tassels, rice panicles, sorghum panicles, and wheat spikes. For wheat spikes, Madec et al. [3] proposed a head density estimation approach that is based on Faster R-CNN and uses high-resolution RGB images. Ma et al. [37], in their work on EarDensityNet, integrated filtering pyramid blocks and dilated convolutions into transfer learning frameworks to enhance the details extracted from high-resolution ground-captured images. This method effectively counters the performance decline usually associated with lower-resolution UAV images. For maize tassel counting, Lu et al. [8] developed TasselNet, which uses random sampling of images to create localized corn images and then generates a regression density map of these images. Zheng et al. [9] introduced MLAENet to address the multiscale challenges in maize tassel counting, which utilizes dilated convolutions to preserve spatial information and expand the receptive field while retaining the resolution of the feature maps. Several methods have been proposed for counting rice panicles. For rice panicle counting, Chen et al. [27] employed refined feature fusion (RFF), which uses multiscale convolutions to produce features at each layer and feature pyramid fusion to integrate the most suitable features across different layers. Liu et al. [32] designed SFC${}^{2}$Net for feature fusion by exploiting a feature pyramid network (FPN) to increase the diversity of receptive field sizes within the last layer of feature maps, providing a multiscale technique for rice counting. Oh et al. [29] established a correlation between the areas of sorghum heads and their counts, utilizing 11 morphological features derived from RGB image segmentation. This model facilitates the generation of density map regression counts for UAV-captured images of sorghum heads.

However, existing methods for counting cereal crops via density maps primarily develop models specific to one type of crop head. Unfortunately, a model trained on a single crop fails to generalize to different crop types because of within-class variance, necessitating specific annotated samples when training a model to count different crops. This paper presents a unified counting model based on few-shot learning for counting various types of crop heads without extensive annotation.

### Few-shot counting

Few-shot learning-based counting methods offer a promising avenue for mitigating the costs associated with iterative training processes [33,38]. These methods are predicated on discerning object counts by analyzing the similarity between query and support images, thereby eliminating the necessity for repeated fine-tuning [39]. Pioneering efforts in this domain were initiated by the generative matching network (GMN) [40], which conceptualizes class-agnostic counting tasks as matching problems, thus exploring the utility of self-similarity algorithms. Building on this foundation, Ranjan et al. [33] advanced the field by employing region of interest (ROI) pooling for density map prediction and introduced the FSC-147 dataset, which is a novel resource for class-agnostic counting. Subsequent developments have bifurcated into 2 primary streams: one focused on leveraging sophisticated visual backbones such as vision transformers (ViT) to enhance the capability of feature extraction [41], and the other on augmenting the proficiency of a model in matching support and query images [35,36,42]. However, the intricate background complexity caused by the presence of leaves, overlapping instances, and variations in head size in cereal crop images makes establishing relationships between support and query images challenging. Thus, applying FSC methods directly to the task of counting crop heads could result in unsatisfactory performance.

## Method

**Fig. 3.**
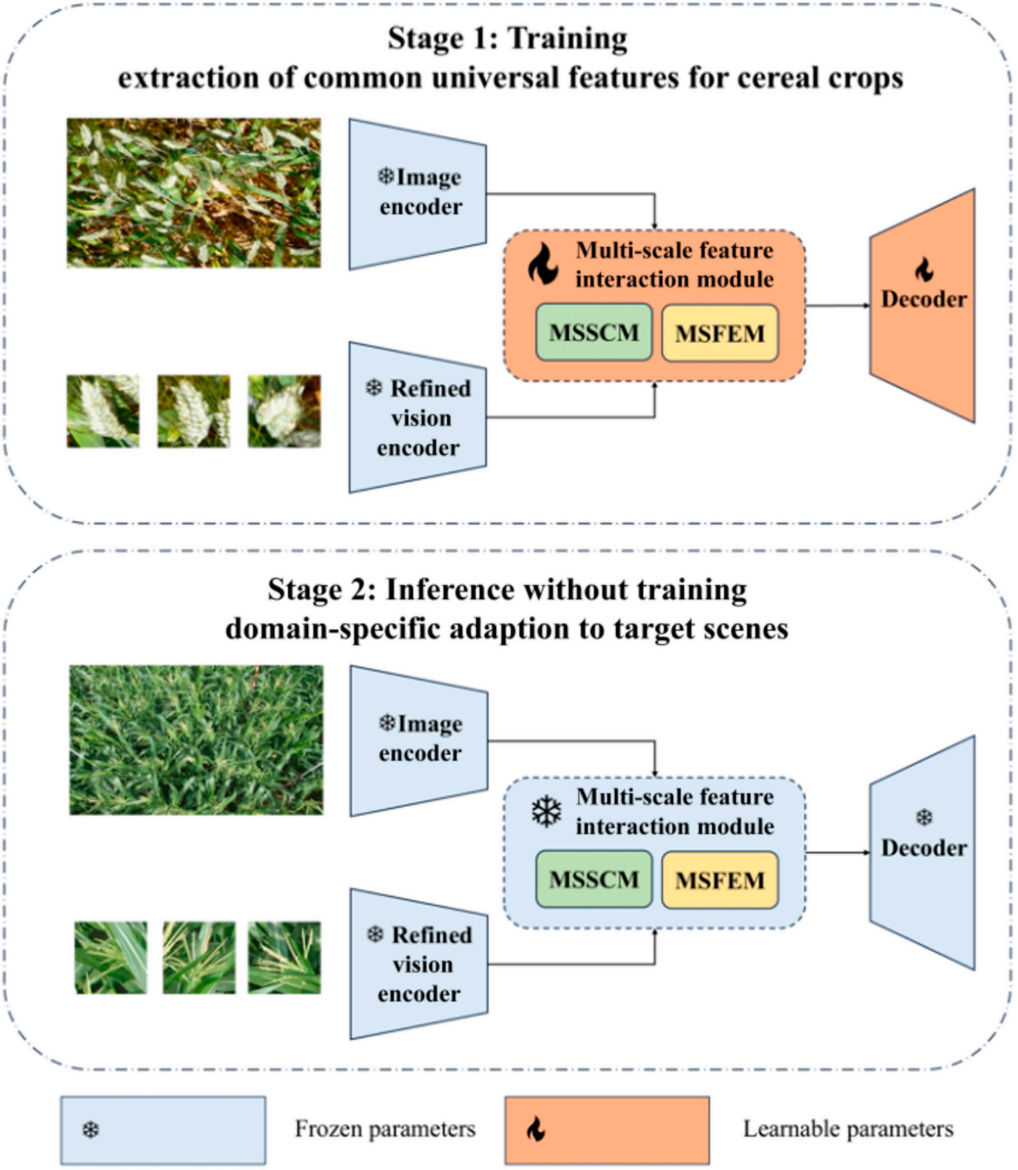
Overview of the CHCNet framework depicting the 2-stage process of training and inference. The model learns common cereal crop features during training and adapts to domain-specific characteristics during inference without additional training, enabling direct counting across diverse cereal crop types.

The proposed CHCNet (Counting Heads of Cereal Crops Net) is structured primarily as shown in Fig. 3 and comprises 2 stages. During the first stage, latent feature mining is performed to learn the common feature representations of cereal crops. In the second stage, inference is conducted without any training, wherein domain-specific features of the target crop are acquired from the selected exemplars (support images) to facilitate the crop counting process. The architectural framework remains consistent across both stages. The image encoder and the RVE are used to extract features from query images and support images, respectively. Next, a multiscale feature interaction module, including a multiscale similarity comparison module (MSSCM) and a multiscale feature enhancement module (MSFEM), automatically learns the relationships between features via a similarity metric. Finally, the decoder generates density maps on the basis of cereal crop feature maps and conducts regression counting. The weight parameters of the multiscale feature interaction module and decoder are trained in stage 1 and then frozen for stage 2. The weight parameters of the image encoder and RVE are frozen in both stages, which allows for offline implementation.

### Refined vision encoder

The proposed CHCNet introduces prototype vectors generated by the RVE to represent counting targets within support images. Typically, these prototype vectors are derived from support images by a combination of ROI pooling followed by average or max pooling. Advancements such as LOCA [36] have extended this groundwork by incorporating attention mechanisms to aid in extracting the prototype vectors. In the context of cereal crop counting, however, support images defined by object detention bounding boxes lead to extraneous background inferences. This significantly hampers object prototype extraction, potentially reducing counting performance. Furthermore, in our specific context concerning cereal crops, which are often characterized by elongated panicles, multiple panicles may be required to represent a single crop entity. This scenario, where detection boxes encompass large areas despite the relatively small size of the actual object, results in a predominance of background within the support images, further exacerbating the issue of background interference. To address this issue, we propose an RVE that uses masks instead of bounding boxes, which helps distinguish target crop heads from the background. Compared with bounding boxes, masks offer superior detail, distinctly delieating object boundaries and shape information. The recently introduced foundational segmentation model, the SAM [43], can segment objects using only boxes or points as cues. Moreover, it is adept at segmenting all the objects in an image efficiently in one-shot or few-shot modes. Building on this idea, we propose leveraging the bounding boxes from support images as prompts for the SAM to generate crop head masks.

Following this, we extract features aligned with the mask locations from the embedded features via the ViT [44], which functions as the image encoder for both support and query images. To improve generalizability, we utilize a pretrained DINOv2 feature extractor for ViT embedding. These chosen features subsequently undergo average pooling to generate the ultimate prototype vectors. Given a support image $I_{S}$, the refined mask image $M_{R}$ is generated as

$$M_{R}=\mathrm{SAM}\left(I_{S}\right),$$ (1)

where SAM(⋅) denotes the use of the SAM [43] to segment the support image $I_{S}$

The ViT embedded features $F_{P}$ of a query image $I_{Q}$ are given by

$$F_{P}=\mathrm{ViT}\left(I_{Q}\right),$$ (2)

where ViT(⋅) denotes the use of the ViT encoder [44] to extract features. The features of the refined mask $F_{S}$ are computed as follows:

$$F_{S}=F_{P}\cdot M_{R}.$$ (3)

**Fig. 4.**
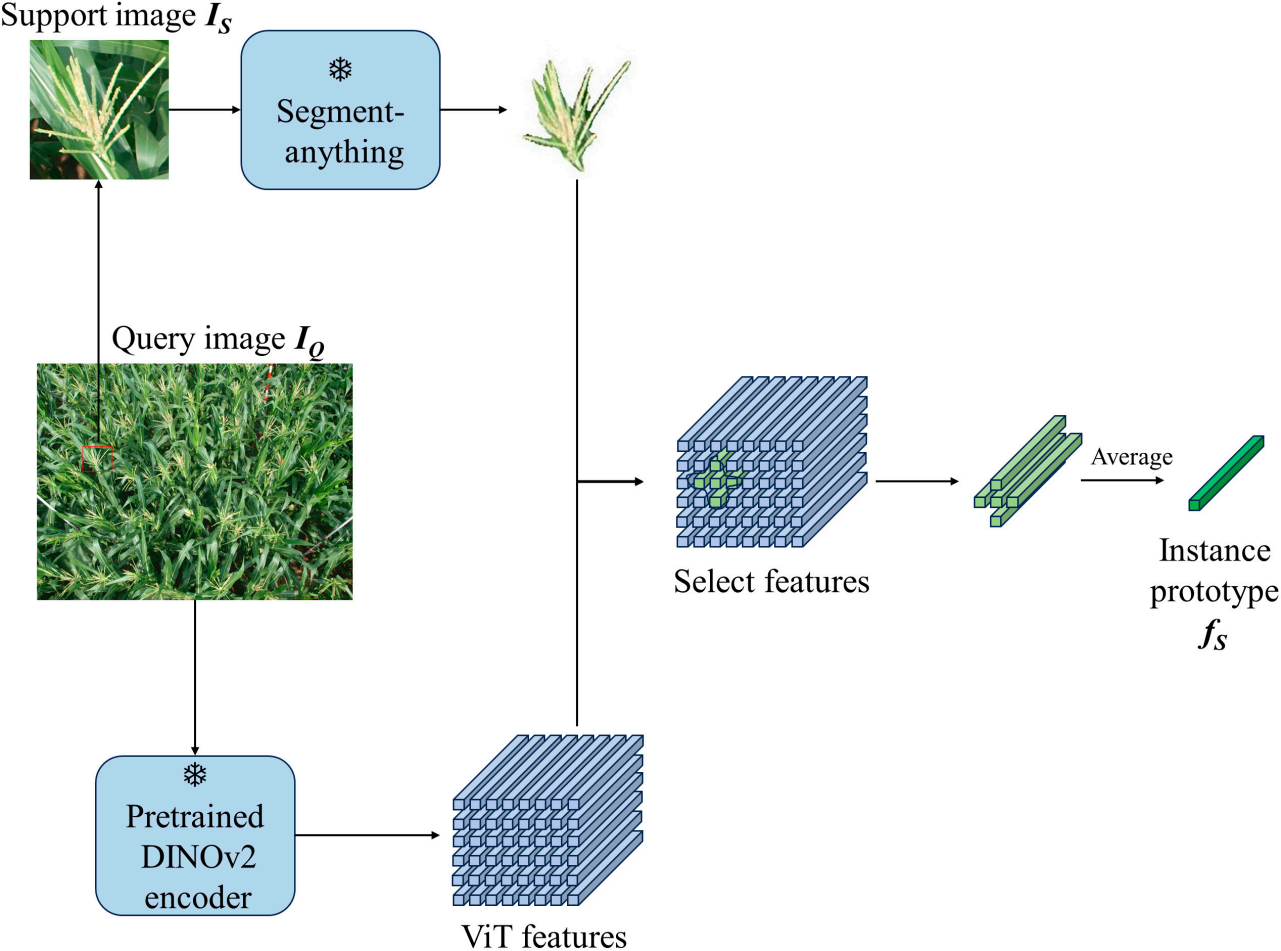
Overall workflow of RVE.

To facilitate subsequent multiscale analysis, the support image features $F_{S}$ are pooled into prototypes $F_{S_{k}}$ at 3 different scales (i.e., small, medium, and large). Here, *k* represents the different scales, where different sizes of average pooling operations are used. Figure 4 illustrates the prototype construction process.

### Multiscale feature interaction module

The multiscale feature interaction module, which is a pivotal component of our proposed architecture, is intricately designed to enrich feature representations through a twofold process. First, the MSSCM performs the task of comparing features across multiple scales. By leveraging the refined prototypes of various scales generated by the RVE, the MSSCM employs a sliding window to perform point-by-point comparisons between these multiscale prototypes and the query image. This comparative analysis results in the generation of similarity maps, which offer a unique scale-specific perspective on feature resemblance.

Next, the MSFEM is employed to integrate the similarity map generated by the MSSCM with the original supporting image features to reconstruct a similarity feature map containing spatial information. The integrated similarity feature maps undergo a cascaded attention mechanism before being added to the query image for feature enhancement. In this mechanism, spatial attention and multiscale convolutional attention are employed to enhance the discriminative capability of feature representation.

#### Multiscale similarity comparison module

Similarity maps provide enhanced representations of the relationships between support and query images. Conventional methods typically employ fixed-size prototypes for similarity comparison [33,45], which encounter challenges when dealing with the varying sizes of crop heads in our specific contexts.

**Fig. 5.**
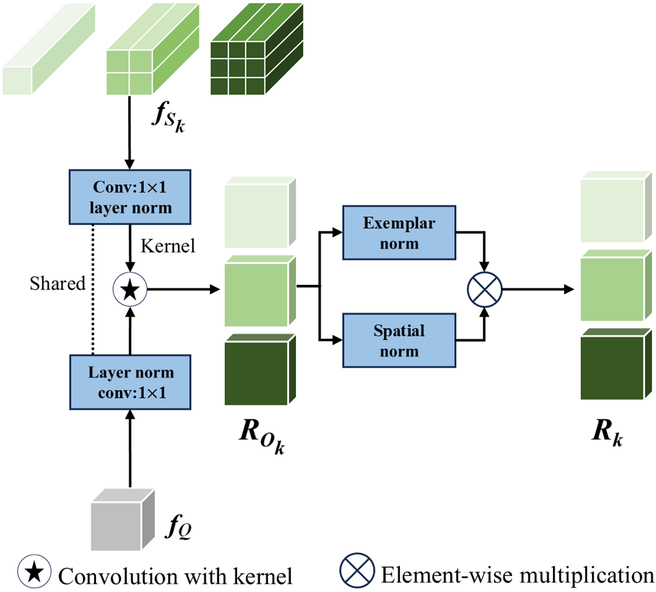
Overall workflow of the MSSCM

To address this challenge, we perform similarity comparisons between prototypes from the RVE and the query image features across different scales, which enhances the ability of the MSSCM to recognize objects of different sizes. Pooling operations are performed using 3 different kernel sizes, resulting in the generation of 3 prototypes representing varying scales. Small-scale prototypes $f_{S_{1}}$ retain the original feature scale and potentially increase feature channel nonlinearity and depth. Medium-scale prototypes $f_{S_{2}}$ integrate information from neighboring pixels to capture local spatial patterns. Large-scale prototypes $f_{S_{3}}$ expand this framework by including larger local regions to incorporate more comprehensive spatial contexts, facilitating comparisons from a broader viewpoint. These acquired multiscale prototypes enable the model to leverage multilevel spatial information, thereby enhancing its adaptability across different visual tasks. We represent the query image features as $f_{Q}\in {\mathbb{R}}^{C\times H_{Q}\times W_{Q}}$ and the prototype features as $f_{S_{k}}\in {\mathbb{R}}^{N\times C\times H_{S}\times W_{S}}$, where C is the number of channels in the features, N is the number of support images, H is the height of the features, and W is the width of the features. The MSSCM performs feature mapping, convolutional comparison, and normalization, as shown in Fig. 5.

Feature mapping: Initially, the 3 different-sized prototypes $f_{S}$ and the query image $f_{Q}$ are mapped to the same embedding space via a combination of a 1×1 convolution kernel and layer normalization. This ensures that they have the same distribution.

Convolutional comparison: Using the 3 prototypes as convolution kernels to convolve with the query image $f_{Q}$ generates initial similarity maps $R_{O_{k}}$ via pointwise feature interaction, i.e.,

$$R_{O_{k}}=\text{conv}\left(g\left(f_{Q}\right),\text{kernel}=g\left(f_{S_{k}}\right)\right)\left(k=1,2,3\right),$$ (4)

where *g*() denotes the combination of the 1×1 convolutional layer and layer normalization in feature mapping.

Normalization: The initial similarity maps are then normalized via the exemplar norm $R_{\mathrm{EN}}$ and spatial norm $R_{\mathrm{SN}}$, ensuring a consistent scale and distribution of similarity maps R, as follows:

$$R_{{\mathrm{EN}}_{k}}={\text{softmax}}_{\text{dim}=0}\left(\frac{R_{O_{k}}}{\sqrt{H_{S}W_{S}C}}\right)\left(k=1,2,3\right),$$ (5)

$$R_{{\mathrm{SN}}_{k}}=\frac{\text{exp}\left(R_{0_{k}}/\sqrt{H_{S}W_{S}C}\right)}{{\text{max}}_{\text{dim}=\left(2,3\right)}\left(\text{exp}\left(R_{O_{k}}/\sqrt{H_{S}W_{S}C}\right)\right)}\left(k=1,2,3\right),$$ (6)

where *dim* = 0 signifies the dimensionality associated with the supporting image samples; $\dim=\left(2,3\right)$ denotes the height and width, respectively, of the extracted features; ${\text{softmax}}_{\text{dim}}\left(\cdot \right)$ is the Softmax layer applied along a specific dimension; and ${\text{max}}_{\text{dim}}\left(\cdot \right)$ finds the maximum value from the given dimensions. The final similarity maps $R_{k}$ are given by

$$R_{k}=R_{{\mathrm{EN}}_{k}}⊗R_{{\mathrm{SN}}_{k}}\left(k=1,2,3\right),$$ (7)

where ⊗ denotes elementwise multiplication.

Finally, 3 similarity maps $R_{k}$ across various scales are obtained for more comprehensive similarity learning between the support and query images. This multiscale strategy enhances the robustness of the model to variations in object size within the images, thus enhancing its overall counting performance in our complex and multiobject scenarios.

#### Multiscale feature enhancement module

**Fig. 6.**
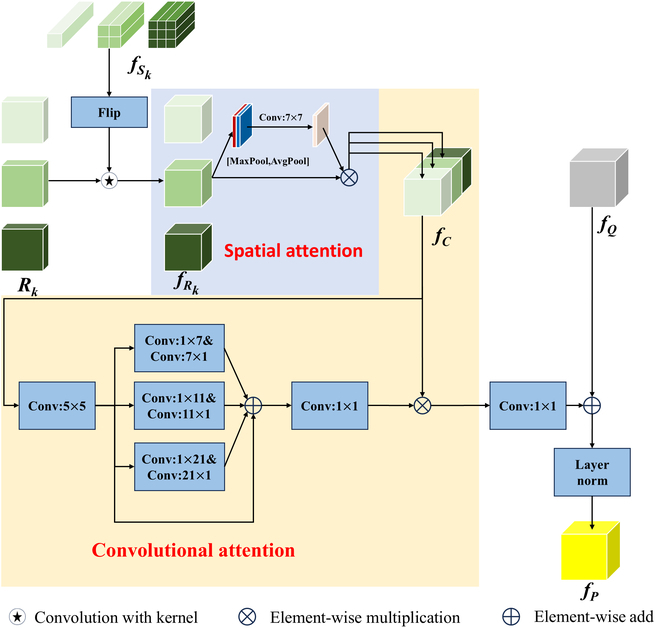
Overall workflow of the MSFEM.

The MSSCM produces similarity maps across 3 different scales to provide similarity information. However, these maps fail to capture diverse spatial properties. Thus, they serve as guidance for obtaining similarity feature maps that include both similarity and spatial information at 3 different scales, i.e., in the multiscale feature enhancement (MSFEM) module. The specific workflow of the MSFEM is illustrated in Fig. 6 and is divided into the following 4 steps.

Converting similarity maps to similarity feature maps: This step uses flipped prototypes as convolution kernels to convolve with similarity maps at 3 different scales. This process generates 3 similarity feature maps $f_{R_{k}}$ at different scales, guided by the similarity maps $R_{k}$ and the support image features $f_{S_{k}}$. The flipping ensures that the similarity feature maps $f_{R}$ maintain the spatial structure of $f_{S_{k}}$ as

$$f_{R_{k}}={\text{sum}}_{\text{dim}=0}(\text{Conv}\left(R_{k},\text{kernel}=\text{Flip}\left(f_{S_{k}}\right)\right)\left(k=1,2,3\right),$$ (8)

where ${\text{sum}}_{\text{dim}}\left(\cdot \right)$ accumulates the input tensors along specific dimensions.

Multiscale spatial attention: The similarity feature maps at 3 different scales globally compare scales without focusing on the corresponding spatial dimensions. Inspired by the attention mechanism [46], we first apply MaxPool and AvgPool along the channel dimension, concatenate the 2 features, and then apply a 7 × 7 convolution to extract a spatial attention map. The resulting spatial attention map is used to perform elementwise multiplication with the original features, yielding similarity feature maps that are focused on their respective spatial scales:

$$f_{R_{k}}^{\prime }=\sigma \left(\text{Conv}\left(\text{AvgPool}\left(f_{R_{k}}\right);\text{MaxPool}\left(f_{R_{k}}\right)\right)\right)\left(k=1,2,3\right),$$ (9)

where σ denotes the sigmoid function and Conv represents a convolution operation with a kernel size of 7 × 7.

Fusion of multiscale similarity feature maps: The spatially focused similarity feature maps $f_{R_{k}}^{\prime }$ are fused via simple concatenation to obtain $f_{C}$. This is followed by aggregating local information with a 5 × 5 depthwise convolution and then capturing the multiscale context with multibranch depthwise convolutions. The multibranch deep convolutions employ convolutional kernels of sizes 1 × 7 and 7 × 1 for the first branch, 1 × 11 and 11 × 1 for the second branch, and 1 × 21 and 21 × 1 for the third branch. Finally, the interchannel relationships are modeled with 1 × 1 convolutions. By employing multiscale convolutional attention, the model adaptively selects the weights of similarity feature maps across 3 different scales. After a 1 × 1 convolutional layer for dimension reduction, we obtain a fused similarity feature map $f_{C}^{\prime }$ across 3 scales.

Feature enhancement: The fused similarity feature map is added to the initial feature map $f_{Q}$ generated by the similarity comparison module for feature enhancement, resulting in an enhanced feature map $f_{P}$. This enhanced feature map $f_{P}$ is then fed into the similarity comparison module as the input feature map $f_{Q}$ for N iterations of feature enhancement to produce a feature map that has been enhanced multiple times on the basis of similarity guidance as

$$f_{P}=\text{LayerNorm}\left(f_{Q}+h\left(f_{C}^{\prime }\right)\right),$$ (10)

where $h\left(\cdot \right)$ is implemented with 2 convolutional layers and LayerNorm denotes the layer normalization. The module efficiently integrates and enhances features across diverse spatial positions in similarity feature maps across various scales.

### Decoder

**Fig. 7.**
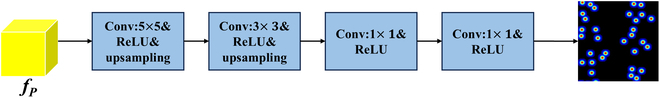
Overall workflow of the decoder.

Following the MSFEM, a regression head is utilized to convert features into density maps. Unlike previous approaches [33,34,40,47], as shown in Fig. 7, our decoder is simpler and comprises a series of convolutional layers, ReLU activation functions, and bilinear upsampling.

### Training loss

Consistent with prior work, our approach involves generating ground-truth density maps $D_{\mathrm{GT}}$ from annotated coordinates via Gaussian smoothing with an adaptive window. Our model is trained by utilizing the mean square error (MSE) loss as follows:

$$ℒ=\frac{1}{M}\left|\right|D-D_{\mathrm{GT}}{\left|\right|}_{2}^{2},$$ (11)

where *M* is the number of samples in the mini-batch and D denotes the predicted density map.

## Experimental Results

### Datasets

**Fig. 8.**
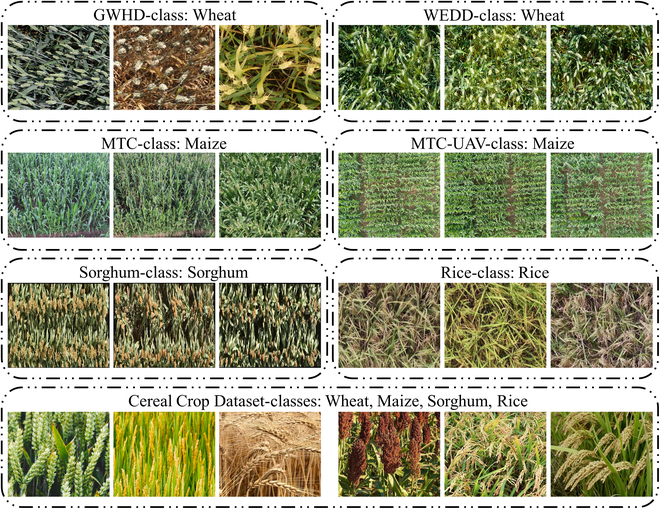
Example images from datasets used.

We utilized 6 publicly available datasets in addition to one dataset that we constructed ourselves. To better match our scenario, images from these datasets where the number of objects to be counted is fewer than 5 were excluded. Examples from each dataset are shown in Fig. 8, and Table 1 shows the statistics of the datasets used.

**Table 1.** Statistics of the datasets used in the one-to-all setting

Set	Class	Source	#Images
Train	Wheat	GWHD [48]	4,627
Val	Wheat	GWHD [48]	1,704
Test	Wheat	WEDD [3]	236
Test	Maize	MTC [8]	310
Test	Maize	MTC-UAV [49]	2,904
Test	Sorghum	Sorghum [50]	1,300
Test	Rice	Rice [51]	2,169
Test	Wheat, maize, sorghum, rice	Cereal Crop Dataset	400

GWHD: This dataset, provided by [48], is an object detection dataset for wheat spikes that comprises over 6,000 images with 1,024 × 1,024 pixels and contains more than 300,000 wheat spikes with their corresponding bounding boxes. These images were acquired from 11 countries/regions, covering 44 unique measurement sessions. Given its diversity, we chose this dataset for training and validation. This is because a diverse dataset promotes strong generalization in models, even within the same class during training.

WEDD: This dataset, provided by [3], is a target detection dataset for wheat spikes that consists of 236 high-quality images of 6,000 × 4,000 pixels and includes 30,729 wheat spikes with their corresponding bounding boxes. It was used to evaluate the ability of the proposed model to be generalized within a domain (crop species).

MTC: Provided by [8], this is a dataset for counting maize tassels of 6 varieties and consists of 361 high-quality images with varying resolutions, such as 3648×2736, 4272×2848, and 3456×2304. The dataset comprises 16 independent time series that span most of the growth period of the maize, reflecting the growth and quantity of maize tassels at various stages. As it is a dataset with point annotations, we manually annotated 3 object detection boxes on each image to serve as support images.

MTC-UAV: This dataset, also provided by [49], is for UAV-based counting of maize tassels. It comprises 306 high-quality images of 5472×3648 pixels and includes 70,870 point-annotated maize tassels. Owing to GPU memory constraints, we cropped each image into 12 patches by dividing it into 2 equal parts horizontally and 6 equal parts vertically, which served as support images.

Sorghum: The dataset [50] comprises sorghum panicles collected in the summer of 2015–2016 at Hermitage in Queensland, Australia ($28.21^{\circ }$S, $152.10^{\circ }$E, at an elevation of 459 m), with an average planting density of 115,000 plants per hectare. There were 2 rows in each plot, which were 5 m in length. The experiment was conducted via solid row configurations with a row spacing of 0.76 m, where the distance between 2 adjacent plots was 1 m. Specific rotations were applied to generate different field orientations. In total, the dataset provides 1,440 high-quality images of sorghum panicles, and 28,825 sorghum panicles were extracted from the original images.

Rice: This dataset contains images of rice panicles from Bangladesh [51] and is intended to facilitate rice yield estimation through the application of object detection techniques. The data collection involved drone imaging, which yielded 2,193 original images and 5,701 enhanced images, with annotations for rice spikes. This dataset is notable for its size and detail and for supporting agricultural research, particularly in developing deep learning models for crop management and yield prediction.

Cereal Crop Dataset: We created a mixed dataset of images of 4 types of cereal crops: wheat, maize, sorghum, and rice. Initially, we sourced publicly available and free images of these cereal crops from the internet. We then cropped these images to retain countable parts while removing background areas that are blurred and thus difficult to annotate and count. We subsequently annotated these images via a small sample dataset approach, marking individual crop heads with point annotations and selecting 3 types of cereal crops in each image for bounding box annotations. Our dataset comprises 86 wheat spike images, 60 maize tassel images, 89 sorghum panicle images, and 165 rice panicle images, with a total of 10,904 point annotations of cereal crops and 1,200 bounding box annotations.

### Experimental setup

#### Implementation details

Each query image and its corresponding feature map had dimensions of 512×512 and 128×128, respectively. For the support images, prototype dimensions were meticulously defined at 3 distinct scales, namely, 1×1,3×3 and 5×5, as denoted by k=1,3,5 in Eq. 4 of the RVE module. We reshaped the dimension of the projected features to 256. Training was systematically conducted via the Adam optimizer across 200 epochs, with a batch size of 1. The initial learning rate was 2 × 10^−5^, which was decremented by a factor of 0.25 every 80 epochs to optimize the learning curve. In the proposed CHCNet, each image was annotated via 3 bounding boxes and served as a support image. The support images used in each learning iteration remained consistent.

#### Evaluation metrics

Following [20,33,52], the mean absolute error (MAE), root mean square error (RMSE), and counting accuracy were chosen for measuring the performance of the counting methods:

$$\text{MAE}=\frac{1}{M}\sum_{i=1}^{M}\left|C^{i}-C_{\mathrm{GT}}^{i}\right|,$$ (12)

$$\text{RMSE}=\sqrt{\frac{1}{M}\sum_{i=1}^{M}{\left(C^{i}-C_{\mathrm{GT}}^{i}\right)}^{2}},$$ (13)

$$\text{Counting}\;\text{accuracy}=\left(1-\frac{1}{M}\sum_{i=1}^{M}\frac{\left|C^{i}-C_{\mathrm{GT}}^{i}\right|}{C_{\mathrm{GT}}^{i}}\right)\times 100\%,$$ (14)

where M is the number of query images and $C^{i}$ and $C_{\mathrm{GT}}^{i}$ are the predicted and ground-truth counts, respectively, of the ith query image.

### Comparisons with state-of-the-art methods

#### One-to-all transfer setting

To test the generalizability of the proposed CHCNet, we employed a one-to-all setting, i.e., training on a single type of cereal crop and testing it directly on others without any training. Leveraging the diversity and richness of the GWHD dataset, CHCNet facilitated the learning of universal features among cereal crops. We conducted training and validation on wheat and direct testing on other cereal crops, such as maize, sorghum, and rice, alongside evaluations on the WEDD dataset to ascertain the domain generalization performance of the model, as detailed in Table 2.

**Table 2.** Quantitative results of the one-to-all experiment, where * denotes that the model directly employed the checkpoints trained on the FSC147 dataset as presented in the original paper. The best performances are in bold font, and the second best performances are in italic font.

Metric	Scheme	Method	WEDD	MTC	MTC-UAV	Sorghum	Rice	Cereal Crop Dataset
MAE	Few-shot	FamNet [33]	43.82	55.93	22.65	51.81	*9.36*	30.21
		BMNet [35]	26.72	81.67	16.81	42.57	10.83	25.13
		BMNet+ [35]	25.88	95.38	10.94	41.59	12.71	24.01
		SAFECount [34]	24.78	18.13	11.05	60.21	11.35	22.41
		LOCA [36]	20.76	48.08	41.06	*15.08*	20.80	*20.57*
		CounTR [41]	24.33	*14.79*	*10.29*	20.17	12.58	21.20
		FamNet* [33]	133.39	42.18	33.49	46.33	16.18	58.72
		BMNet+* [35]	80.82	132.12	98.67	56.27	14.07	103.31
		SAFECount* [34]	190.39	120.94	220.80	88.10	77.19	121.38
		LOCA* [36]	328.87	319.87	170.70	106.11	91.28	114.17
		CHCNet (ours)	*22.34*	**9.96**	**9.38**	**13.94**	**7.94**	**15.62**
	Specific-Object	MCNN [20]	339.29	56.41	48.20	77.20	9.95	154.82
		CSRNet [53]	119.42	22.63	13.47	80.46	20.10	141.24
	Zero-shot	Clip-count* [54]	58.97	347.83	161.29	34.20	80.92	50.83
RMSE	Few-shot	FamNet [33]	46.15	65.06	26.93	74.19	*11.03*	36.73
		BMNet [35]	31.76	95.83	21.65	47.70	12.16	31.80
		BMNet+ [35]	32.94	112.28	14.19	47.18	13.70	30.52
		SAFECount [34]	31.06	22.87	14.35	63.08	15.09	29.47
		LOCA	**24.55**	54.55	46.74	*19.12*	24.95	*28.11*
		CounTR [41]	26.73	*18.94*	*13.92*	22.37	12.90	29.24
		FamNet* [33]	136.56	49.49	61.93	64.89	22.45	64.43
		BMNet+* [35]	120.75	162.92	122.17	60.79	16.36	110.90
		SAFECount* [34]	210.83	144.47	247.08	110.65	86.76	130.65
		LOCA* [36]	349.33	344.80	190.16	119.21	140.29	128.93
		CHCNet (ours)	*25.41*	**14.30**	**12.11**	**17.12**	**10.20**	**24.73**
	Specific-Object	MCNN [20]	358.15	83.58	52.19	79.16	11.48	193.21
		CSRNet [53]	148.65	29.81	17.11	82.34	22.66	157.01
	Zero-shot	Clip-count* [54]	64.35	363.99	164.14	37.59	80.54	61.22

The quantitative results presented in Table 2 show the superiority of our CHCNet model over current FSC approaches, including FamNet, BMNet, SAFECount, and LOCA. Moreover, comparisons with specific-object and zero-shot counting methods reveal their inadequacies in direct testing without fine-tuning, particularly for cereal crops. Notably, leveraging checkpoints from an FSC approach trained on the FSC147 dataset across various object categories did not improve the ability of the model to generalize to cereal crops.

**Fig. 9.**
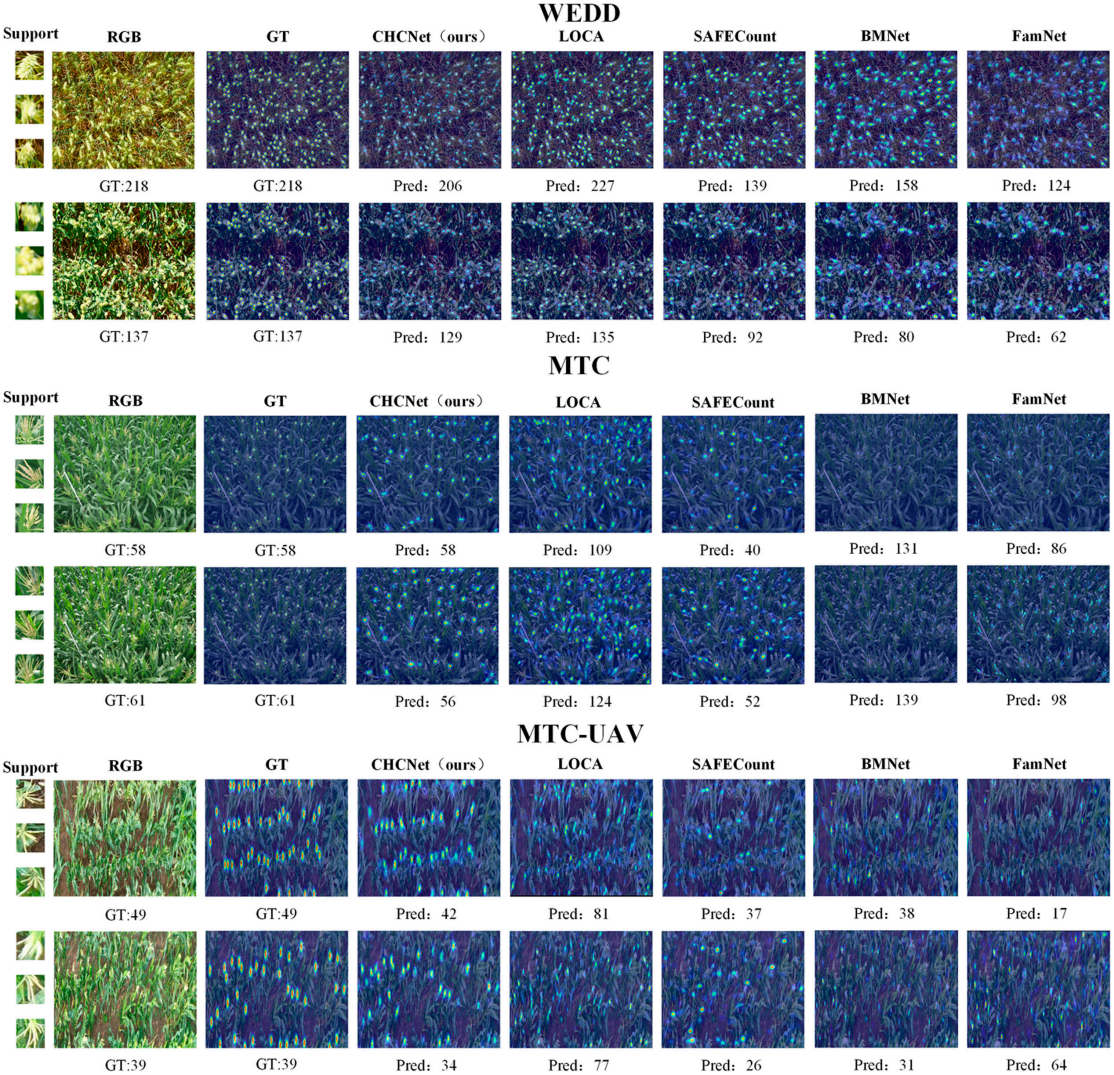
Qualitative results of WEDD, MTC, and MTC-UAV in the one-to-all experiment in the 3-shot case. From left to right: support images, RGB images, query images overlaid by the ground-truth density map, and predicted density maps generated by our method, LOCA [36], SAFECount [34], BMNet [35], and FamNet [33]. The numbers below are the counting results.

**Fig. 10.**
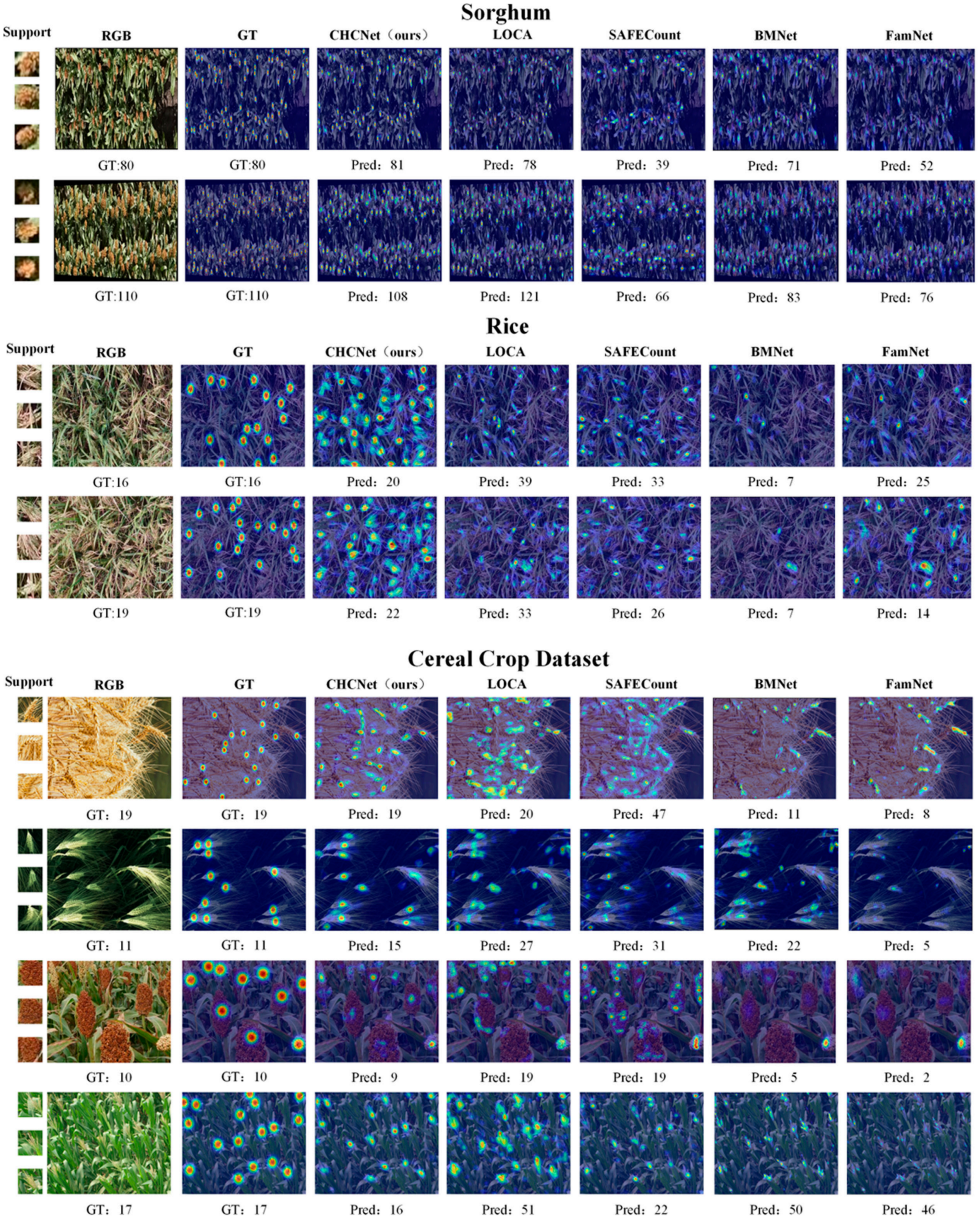
Qualitative results of the one-to-all experiment on the Sorghum, Rice and Cereal Crop datasets in the 3-shot case. From left to right: support images, RGB images, query images overlaid by the ground-truth density map, and predicted density maps generated by our method, LOCA [36], SAFECount [34], BMNet [35], and FamNet [33]. The numbers below are the counting results.

**Fig. 11.**
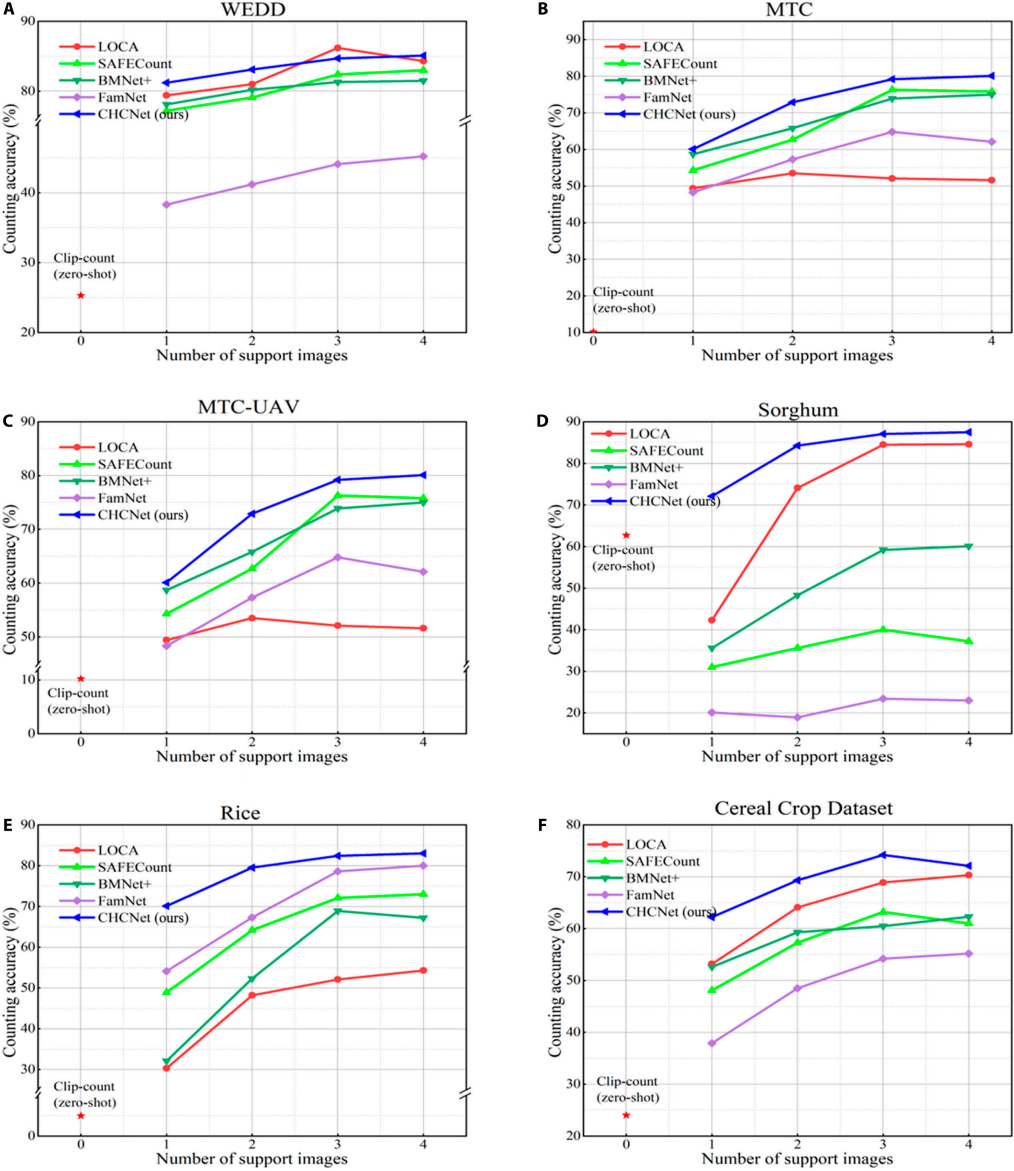
Line plots depicting the counting accuracy of different methods under various shot scenarios across datasets: (A) WEDD, (B) MTC, (C) MTC-UAV, (D) Sorghum, (E) Rice, and (F) Cereal Crop.

Figures 9 and 10 show the qualitative results and comparisons with other FSC methods. The proposed CHCNet exhibits enhanced generalization and localization capabilities for cereal crops, effectively distinguishing individual heads within dense clusters, thereby facilitating accurate counting. To further validate the generalizability of the model, we also assessed the counting accuracy of CHCNet and other FSC methods under various shot conditions. The quantitative results are depicted as a line graph in Fig. 11. The following paragraphs provide detailed comparisons and analyses for each dataset.

WEDD counting results: In the context of a 3-shot experimental setup, Table 2 shows that our model, while marginally inferior to the best-performing model, LOCA, on this dataset, with increases of 1.58 in the MAE and 0.86 in the RMSE, outperformed the next leading model, SAFECount, reducing the MAE by 2.44 and the RMSE by 5.65. Although slightly below LOCA in terms of domain-specific generalization capabilities, our model still maintained a commendable level of performance. Qualitative analysis from Fig. 9 illustrates that our CHCNet model not only demonstrates superior localization ability compared with LOCA but also exhibits enhanced domain generalization over other FSC models. The trend line shown in Fig. 11 underscores the effectiveness of our method in scenarios with fewer shots, which is a testament to the robust generalization capability of our method, which facilitates adaptation to new target domains with minimal shots.

MTC counting results: On the MTC dataset, as shown in Table 2, our CHCNet model achieved remarkable improvements in performance metrics, reducing the MAE by 8.17 and the RMSE by 8.57 compared with the leading model SAFECount. Furthermore, it significantly outperformed the next best model, CSRNet, with a reduction of 12.67 in the MAE and 15.61 in the RMSE, thereby establishing its superior efficacy over other FSC models on this dataset. The qualitative analysis results in Fig. 9 highlight the exceptional ability of CHCHNet to accurately localize corn ears across various scales in a single image without being impeded by background noise. This is attributed to our advanced multiscale mechanism and the RVE module, which effectively eliminates background interference from supporting images. The trend line in Fig. 11 shows a rapid increase in accuracy from the 1-shot scenario to the 2-shot scenario, with our model outperforming the others in subsequent shots. This illustrates the robust generalization ability of our model, which enables seamless adaptation across diverse target domains.

MTC-UAV counting results: On the MTC-UAV dataset, the challenges extended beyond merely assessing the generalization capabilities of our model to include evaluating its proficiency in recognizing small objects. Table 2 shows the superior performance of our CHCNet model, which achieved a reduction in the MAE of 1.56 and in the RMSE of 2.08 compared with the best-performing model BMNet+. Furthermore, it notably outperformed the next leading model, SAFECount, with a decrease of 1.67 in the MAE and 2.24 in the RMSE, positioning our model at the forefront of current FSC approaches. The qualitative results shown in Fig. 9 underscore the exceptional ability of our model to accurately localize small, densely packed UAV-based images of corn ears while effectively mitigating leaf noise interference. This capability is attributed to the unique multiscale mechanism of our method, which adeptly adapts to and recognizes even the minutest objects. The trend shown in Fig. 11 shows the consistent superiority of our method over other methods across various shot scenarios.

Sorghum counting results: Compared with the MTC-UAV dataset, the Sorghum dataset, which is also UAV based, presents a more challenging scenario with denser and finer-scale subjects. According to Table 2, our model achieved a notable improvement on the Sorghum dataset, reducing the MAE by 1.14 and the RMSE by 2.00 compared with the current best-performing model, LOCA. Moreover, it significantly outperformed the next leading model, BMNet+, with reductions of 27.65 in the MAE and 30.06 in the RMSE, establishing a clear superiority over other FSC models. The qualitative results in Fig. 10 demonstrate the enhanced precision of our model in localizing fine sorghum panicles. They also show the superior ability of our model to distinguish background noise, thereby achieving higher counting accuracy. The trend shown in Fig. 11 demonstrates that our model exhibits commendable generalization capabilities in scenarios with fewer shots. This is attributed to the advanced feature extraction capabilities of DINOv2 and the robust generalizability of our model, which facilitates adaptation to new target domains with minimal reliance on domain-specific features and without the need for extensive retraining.

Rice counting results: The images in the Rice dataset are characterized by their complex background, which challenges even human perception in distinguishing rice and poses a marked test to the generalization capabilities of models. Table 2 shows that our model outperformed the current best-performing model on this dataset, FamNet, by achieving reductions of 1.42 in the MAE and 0.83 in the RMSE. Moreover, our model outperformed the next leading model, MCNN, with decreases of 2.01 in the MAE and 1.28 in the RMSE, thus outperforming all the other FSC models. The qualitative results in Fig. 10 highlight the enhanced accuracy of our model in recognizing rice against complex backgrounds to improve the localization precision and counting accuracy. The trend shown in Fig. 11 underscores the consistent superiority of our model across various shot scenarios, including in minimal-shot settings, where it significantly outperformed other FSC models, which is a testament to the exceptional generalization capabilities of our model.

Cereal Crop Dataset counting results: The Cereal Crop Dataset, which is a proprietary compilation encompassing 4 types of cereal crops, serves as an enhanced tool for assessing the comprehensive generalization performance of models across various crop types. As shown in Table 2, our model significantly outperformed the leading LOCA model on this dataset, reducing the MAE by 4.95 and the RMSE by 3.38. Furthermore, it substantially outperformed the next best model, SAFECount, with reductions of 6.78 in the MAE and 4.74 in the RMSE, thereby establishing its dominance over the other FSC models. The qualitative evaluations shown in Fig. 10 affirm the ability of our model to accurately localize and distinguish among different cereal crop types. This is attributed to the exceptional generalization ability of our model, which is tailored to the unique characteristics of cereal crop domains. The trend lines in Fig. 11 corroborate the consistent superiority of our model over other models across various shot scenarios.

A comparative analysis of these datasets underscores the limitations of other FSC methods, which perform well only on a limited number of datasets. In contrast, our method effectively adapts to the target dataset (domains) through domain-specific features, demonstrating commendable performance across all the datasets used in our work.

#### Cross-validation of cereal crops

In addition to the one-to-all experiment, we conducted cross-validation on cereal crops to further validate the transfer performance across different crop types of our CHCNet. We randomly selected 3 hundred images from each of the 4 cereal crops, with each crop type constituting a fold, as specified in Table 3.

**Table 3.** Composition, source, class, and size of each fold

Fold	Class	Source	#Images
0	Wheat	GWHD [48], WEDD [3], Cereal Crop Dataset	300
1	Maize	MTC [8], MTC-UAV [49], Cereal Crop Dataset	300
2	Sorghum	Sorghum [50], Cereal Crop Dataset	300
3	Rice	Rice [51], Cereal Crop Dataset	300

The quantitative results of the cross-validation on cereal crops, detailed in Table 4, show the marked superiority of our method over the newly proposed counting methods, i.e., SAFECount [34] and LOCA [36], in terms of both MAE and RMSE for all the folds.

**Table 4.** Counting performance with cross-validation. Fold-*i* (*i* = 0, 1, 2, 3) indicates the test set.

Methods	Fold-0		Fold-1		Fold-2		Fold-3		Fold-4	
	MAE	RMSE	MAE	RMSE	MAE	RMSE	MAE	RMSE	MAE	RMSE
SAFECount [34]	25.48	28.23	15.90	18.42	19.71	22.13	24.31	29.11	21.35	24.47
LOCA [36]	19.63	24.51	17.89	15.31	15.31	18.74	19.12	24.21	17.99	21.87
CHCNet (ours)	15.11	18.21	10.12	13.78	13.78	17.42	15.09	18.83	13.53	17.34

#### Ablation study

To verify the effectiveness of the various components of CHCNet, we conducted extensive ablation studies ranging from individual to comprehensive experiments.

Refined feature encoder using the SAM: We conducted ablation studies on the use of the SAM in our model to refine prototypes, comparing it with an alternative approach where the bounding boxes from the support images are directly cropped from the feature maps. Additionally, we compared the performance of the SAM when different sizes of ViT parameters were used. The detailed quantitative results are shown in Table 5. Notably, the absence of the SAM for prototype refinement led to a noticeable drop in performance across various datasets.

**Table 5.** Quantitative comparison between CHCNet using the SAM with different ViT weights and its variant without using the SAM

SAM	WEDD		MTC		MTC-UAV		Sorghum		Rice		CCD	
	MAE	RMSE	MAE	RMSE	MAE	RMSE	MAE	RMSE	MAE	RMSE	MAE	RMSE
✗	23.46	26.72	13.28	16.83	12.21	15.19	18.26	24.47	9.03	13.48	18.81	26.02
ViT-h	22.34	25.41	9.96	14.30	12.11	12.11	13.94	17.12	7.94	10.20	15.62	24.73
ViT-l	22.59	25.53	10.13	14.79	12.46	12.46	14.25	17.84	8.23	10.76	15.89	25.19
ViT-b	22.86	25.61	10.47	15.11	12.64	12.64	14.50	18.02	8.49	10.87	16.04	25.31

**Fig. 12.**
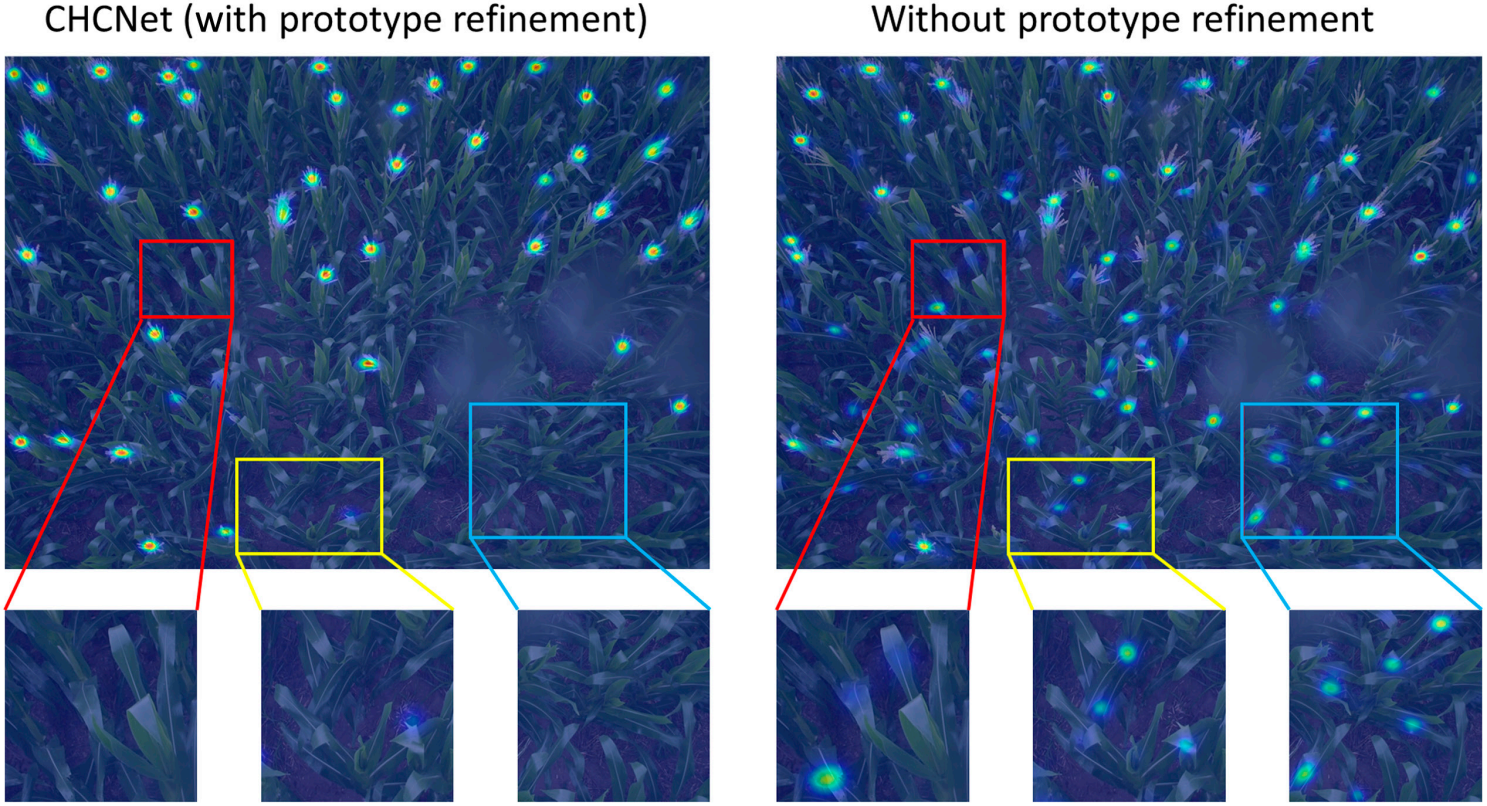
Qualitative comparison between CHCNet and its variant without SAM refinement. The results show that employing the SAM to refine prototypes effectively filters out background noise in support images.

**Fig. 13.**
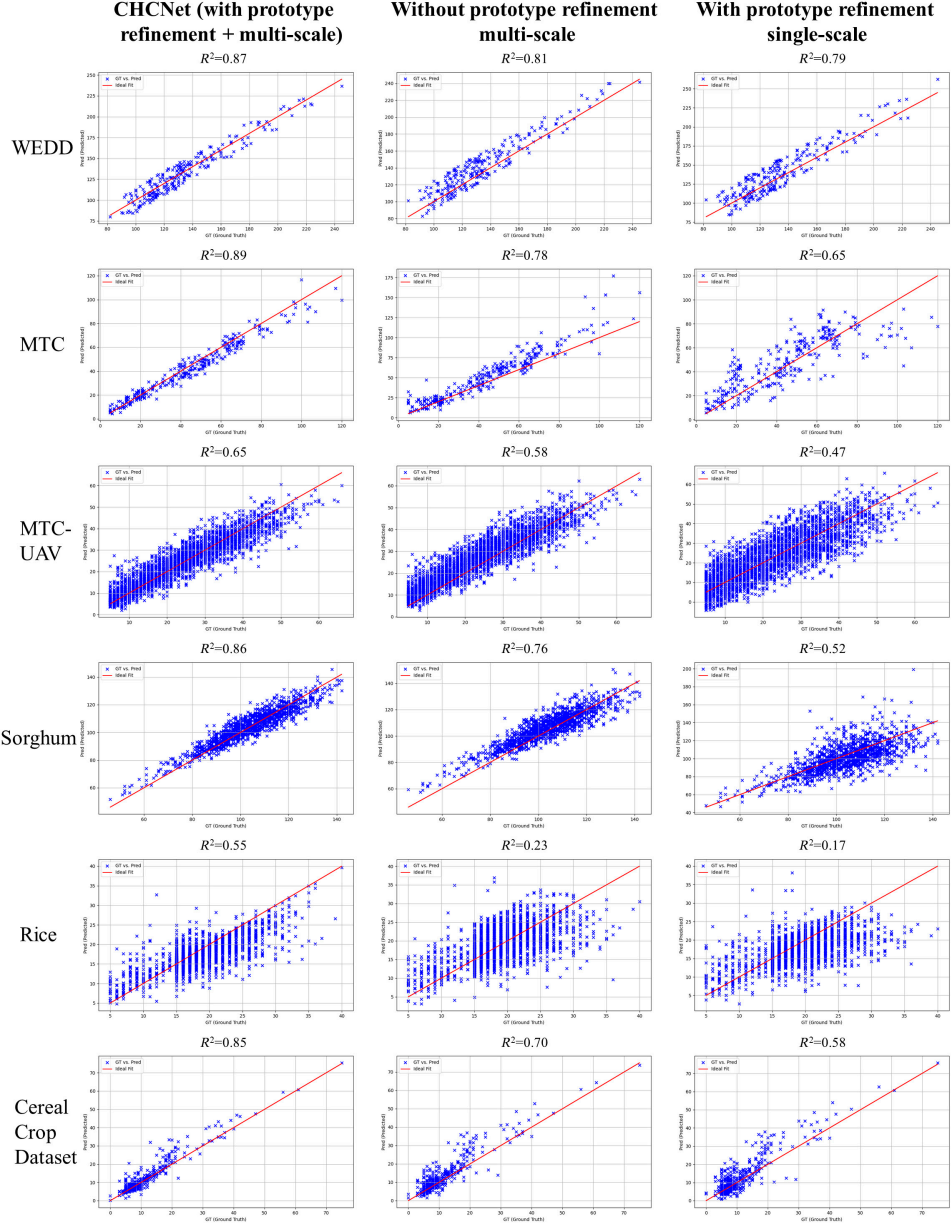
*R*^2^ plots of CHCNet, CHCNet without prototype refinement, and CHCNet with single scale evaluated on various datasets.

Figure 12 presents a visual comparison of density maps with and without the application of the SAM for feature refinement, using the MTC dataset as an example. This shows that without the refinement introduced by the SAM, the model also predicted small counts in the background areas. Figure 13 presents scatterplots of *R*^2^ values for predictions on different datasets with and without feature refinement via the SAM. The plots show that model that does not utilize the SAM for feature refinement tended to overestimate counts more frequently and exhibited lower *R*^2^ values than the model that does use the SAM.

Combining these quantitative and qualitative findings, it becomes clear that utilizing the SAM to refine prototypes significantly reduces the impact of background elements in the support images when employing similarity map-based regression counting models. This RVE module effectively improves the counting accuracy of the model by reducing the number of errors attributed to background noise. Concurrently, the utilization of augmented ViT parameters for the SAM did not yield a substantial enhancement in the model’s accuracy. This finding indicates that while segmentation precision can moderately increase the model’s accuracy, the impact is negligible.

Multiscale module: Ablation studies were conducted to evaluate the effectiveness of the multiscale module in the proposed CHCNet. The alternative to multiscale methods is to use only a 3 × 3 prototype in the similarity comparison module to compare with the query image feature maps. Without multiple scales, subsequent channels for feature fusion and mechanisms for spatial attention are no longer needed, and the similarity feature map is added directly to the original feature map to obtain an enhanced similarity feature map. In the multiscale module design, spatial attention and convolutional attention are employed to fuse multiscale features. Thus, we conducted ablation studies for a single scale and 2 attention mechanisms, with the specific quantitative results shown in Table 6. The table shows that the introduction of a multiscale module significantly enhanced the counting capability for cereal crops. Furthermore, neither applying spatial attention to similarity feature maps at a specific scale nor performing channel attention to similarity feature maps across 3 different scales effectively improved model performance. Only by applying both spatial and convolutional attention could we substantially enhance the multiscale performance of our CHCNet.

**Table 6.** Quantitative comparison between CHCNet and its variant using a single-scale network

Metrice	Scale	Spatial attention	Conventional attention	WEDD	MTC	MTC-UAV	Sorghum	Rice	CCD
MAE	Single	✗	✗	30.47	15.81	16.29	25.48	13.58	21.45
	Multi	✓	✗	24.81	14.29	15.52	15.18	9.06	19.44
		✗	✓	28.34	12.01	11.43	21.79	11.49	19.36
		✓	✓	22.34	9.96	9.38	13.94	7.94	15.62
RMSE	Single	✗	✗	36.59	19.09	19.90	29.37	15.29	27.92
	Multi	✓	✗	27.97	17.43	18.45	19.63	12.91	26.09
		✗	✓	32.17	16.22	14.74	26.60	13.57	25.91
		✓	✓	25.41	14.30	12.11	17.12	10.20	24.73

**Fig. 14.**
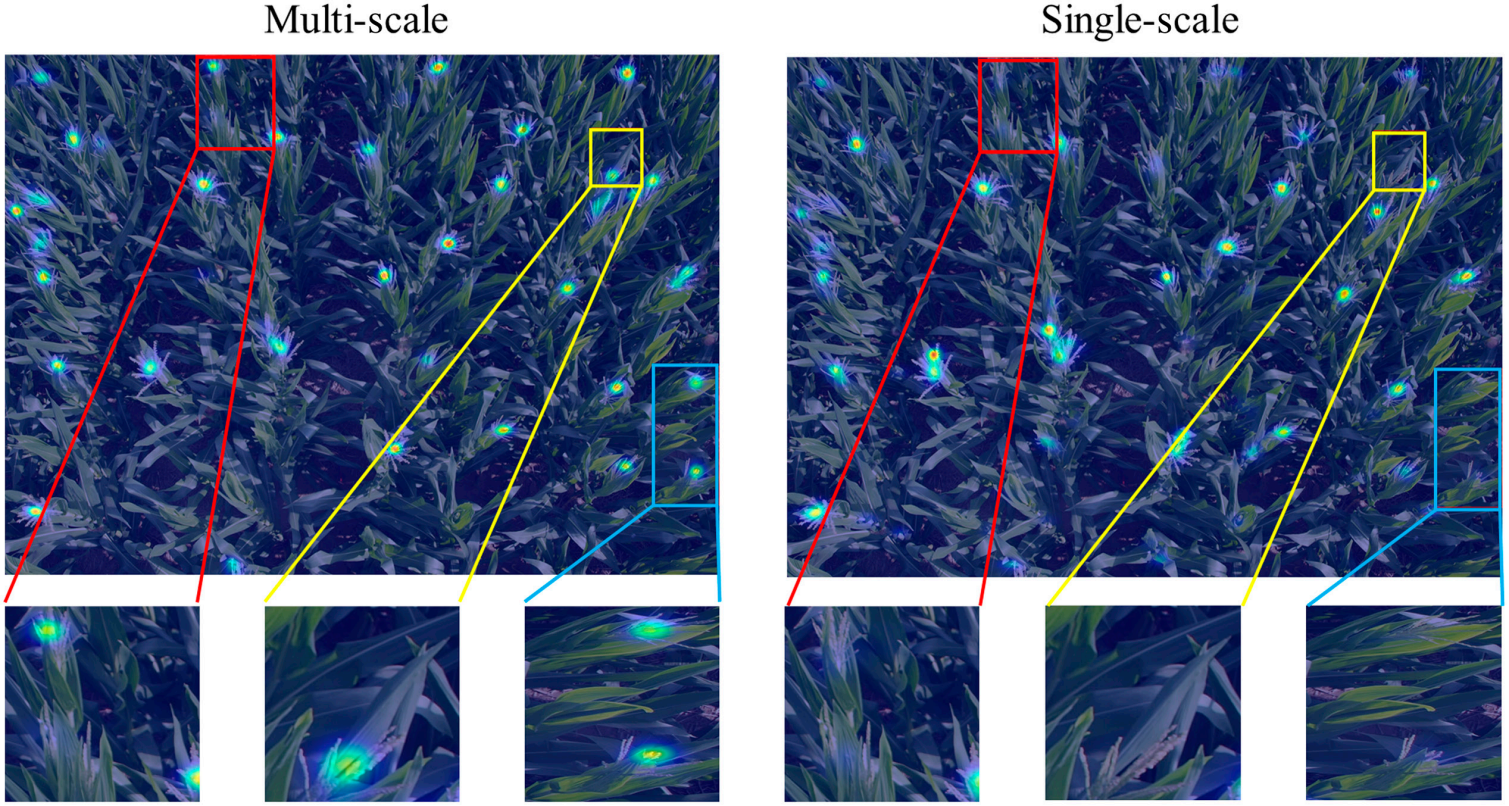
Qualitative comparison between CHCNet and its variant that uses a single-scale network.

To more effectively illustrate the benefits of the multiscale module over the single-scale approach, Fig. 13 presents a comparative visualization of density maps produced by multiscale and single-scale models, with MTC serving as the exemplar. The results indicate that the single-scale model encounters greater difficulty than the multiscale model in detecting objects with important directional and size variation. Figure 14 shows scatterplots of *R*^2^ values for both multiscale and single-scale models across various datasets. In these *R*^2^ plots, the single-scale model exhibits a more dispersed prediction distribution, with predictions further away from the true values and lower *R*^2^ values, indicating less accuracy.

From both the quantitative and qualitative analyses, it can be concluded that the proposed CHCNet, which is equipped with a multiscale module, is adept at predicting objects of varying sizes within images, including very small objects. The multiscale module enables the model to transform support images into similarity feature maps at different scales for comparison with query images. Through the use of channel and spatial attention mechanisms, similarity feature maps from different scales are subsequently integrated to enhance the features. This process significantly improves the ability of our model to predict objects across scales, enhancing its overall prediction accuracy.

Pretrained feature extractor: Using pretrained feature extractors can greatly reduce the training workload of a model and improve its performance. Both DINOv2 and ResNet have pretrained models available. We designed experiments using DINOv2 and ResNet-18 as feature extractors, and Table 7 shows the quantitative results. The experimental results show that, compared with ResNet, which was trained only on ImageNet-1k [55], DINOv2, which was trained on a larger dataset LVD-142M without supervision, performed significantly better. It not only extracts powerful image features but also requires no fine-tuning for downstream tasks, making it suitable as a new backbone for various applications, including our scenario.

**Table 7.** Quantitative comparison between ResNet-18 and DINOv2 as the backbone

Feature extractor	WEDD		MTC		MTC-UAV		Sorghum		Rice		CCD	
	MAE	RMSE	MAE	RMSE	MAE	RMSE	MAE	RMSE	MAE	RMSE	MAE	RMSE
ResNet-18	23.86	30.93	13.12	17.72	12.89	14.70	20.93	24.16	12.44	15.37	19.58	29.10
DINOv2	22.34	25.41	9.96	14.30	9.38	12.11	13.94	17.12	7.94	10.20	15.62	24.73

Computational complexity and efficiency: A comparative analysis of the computational complexity and efficiency of the proposed CHCNet was conducted using input images with a resolution of 512 × 512 pixels. Commonly used metrics, specifically the numbers of parameters and floating-point operations per second (FLOPs), were employed in our experiments, with the results presented in Table 8. As indicated in the table, the proposed CHCNet has nearly half the number of parameters and approximately 5 times fewer trainable parameters than CounTR, which also employs a ViT as its encoder. Moreover, CHCNet demonstrates comparable performance relative to other state-of-the-art methods in terms of parameter count. Although the number of FLOPs of the proposed CHCNet was slightly higher than those of LOCA and CounTR, it achieved important improvements in accuracy across various crop datasets. The complexity and computational efficiency of the 2 designed modules, the MSSCM and MSFEM, are also presented in Table 8. As illustrated in the table, both proposed modules have low parameter numbers and computational complexity.

**Table 8.** Computational efficiency and model complexity analysis using different methods. #Params denotes the number of network parameters, and #GFLOPS refers to the number of FLOPs on a 512×512 input image.

Method/module	#GFLOPS	#Params	
		Total	Trainable
FamNet	55	26M	760K
BMNet+	27	13M	12M
SAFECount	366	32M	20M
LOCA	80	37M	11M
CounTR	91	100M	99M
CHCNet (ours)	120	44M	23M
MSSCM	13	7M	7M
MSFEM	24	10M	10M

## Discussion

### Effectiveness of using SAM

The influence of background noise such as leaves has been discussed for RFF-PC [27], which poses a substantial challenge. A potential solution to this issue involves segmenting and removing irrelevant pixels to mitigate the impact of such noise on density estimation in images. Notable efforts in this direction have been presented with extensive experimentation in [29,56]. A prime example is TasselNetV3 [49], where a local counting network is integrated with a segmentation network to suppress background interference. In this context, the newly proposed vision foundation model, i.e., the SAM, is employed to mitigate background noise due to its powerful segmentation ability, where the selected exemplar bounding boxes are used as the input for the box prompt (as shown in [15]). Benefiting from the SAM, the crop head targets are highlighted, thus enhancing the feature encoding process.

**Fig. 15.**
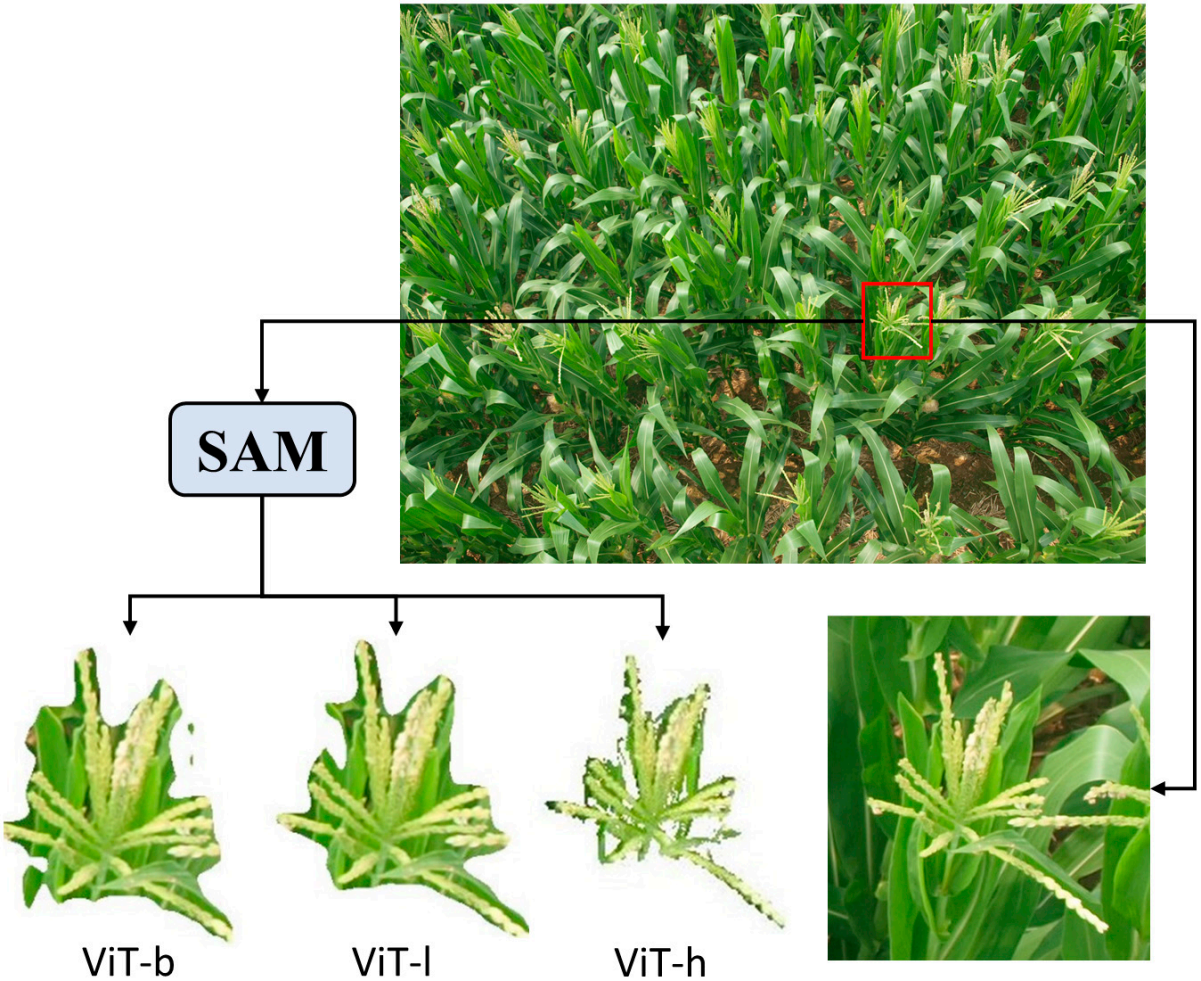
Visual comparison of SAM with different ViT weights and without SAM.

However, the segmentation method for support images in the proposed architecture is not limited to the SAM. Any segmentation method capable of segmenting target objects, such as threshold segmentation, can be used. The main reason for using the SAM is its ability to segment anything without the need to set thresholds for specific classes or train a new segmentation network, which aligns well with the fundamental concept of few-shot learning and practical scenarios. As demonstrated in Table 5 through ablation experiments on the SAM’s ViT weights, improvements in segmentation accuracy can enhance the model’s accuracy to some extent, but the effect is minimal. Furthermore, from the segmentation results for spike crops by the SAM under different ViT parameters shown in Fig. 15, it is evident that the SAM’s ability to segment spike crops is somewhat lacking and not perfect. However, we should focus not on the background that was not segmented but rather on the background that was segmented out, as it effectively filtered out most background noise. Therefore, the ability to segment anything is more important than segmentation accuracy.

### Effectiveness of using a multicolumn structure

Although transitioning traditional FSC from a single-column structure to a multicolumn structure introduces complexity, it also helps the model better adapt to different scale variations and generalize from single-scale support images to multiscale query images. The multiscale structure broadens the model’s perspective and generalization capability, while segmenting support images to filter out background noise enhances the model’s depth perception. When combined, multiscale and feature refinement enable the model to better handle occluded spike crops of smaller scales and reduce background noise.

### Label efficiency

The number of crop heads is a critical plant phenotype, and accurately and efficiently counting them is an important task in the area of plant phenotyping. This process can significantly benefit breeding programs and yield estimation. Thus, substantial research efforts have been dedicated to addressing the problem of crop head counting, with wheat, maize, rice, and sorghum being the primary research targets [3,8,9,27,29]. For example, Madec et al. [3] introduced a head density estimation network for counting wheat spikes via Faster R-CNN with high-resolution RGB images; Zheng et al. [9] proposed MLAENet to address multiscale challenges via dilated convolutions to preserve spatial information and expand the receptive field in the maize tassel counting task; Chen et al. [27] employed RFF-PC to count rice panicles, utilizing multiscale convolutions and feature pyramid fusion to integrate features across layers; and Oh et al. [29] established a correlation between sorghum head areas and counts using 11 morphological features from RGB image segmentation, facilitating density map regression counts for UAV-captured images. However, existing methods are typically tailored to specific types of crop heads. Models trained on a single crop type often fail to generalize across different crop types because of within-class variance, necessitating specifically annotated samples for each new model. In contrast, we propose CHCNet, which pretrains a crop counting model using point-supervised annotations and adapts to different types of crop heads with a few box annotations without additional training. This approach enables efficient and accurate cereal head counting without requiring extensive annotated samples from the target domain, thereby significantly reducing annotation costs. More importantly, this method demonstrates strong transferability to other crop head counting tasks. Researchers can utilize our model for their specific crop head counting needs with only a few bounding box annotations for the heads to be counted. We believe that these advancements could serve as a general tool for crop head counting, providing a robust solution for reducing labeling costs and enhancing the efficiency of crop counting tasks.

### Future research prospects

The proposed CHCNet exhibits promising performance in cross-crop transfer scenarios, positioning itself as a versatile tool for counting various types of crop heads across diverse data acquisition environments. Future research could focus on integrating classification networks to enable the model to perform counting and crop recognition tasks simultaneously. Additionally, pursuing lightweight design strategies may be another avenue for research aimed at further reducing the model’s computational complexity while enhancing efficiency.

## Conclusions

In this work, to devise a model that can uniformly count all types of cereal crops without necessitating extensive training across numerous crop classes, we propose a few-shot learning-based counting method, CHCNet. We engineer an RVE to effectively enhance feature embedding by employing a vision foundation model, namely, the SAM, to focus attention on the salient head target while reducing the effects of complex backgrounds, such as leaves and stems. Furthermore, we propose a multiscale feature interaction module that incorporates similarity learning, which enables the model to automatically learn domain-specific features and similarities between exemplars and image regions across different scales, thereby generating information-rich similarity feature maps. CHCNet demonstrates promising cross-crop performance across various datasets and can be widely applied in various cereal crop counting scenarios. In the future, a more diverse set of samples will be gathered for training, thereby enhancing the counting model’s capabilities. Additionally, the crop recognition module will be integrated to achieve the counting and classification of crops in unseen domains.

## Data Availability

The datasets used in our experiments, including GWHD, WEDD, MTC, MTC-UAV, Sorghum, and Rice, are publicly accessible.

## References

[1] Osco LP, Arruda MS, Gonçalves DN, Dias A, Batistoti J, de Souza M, Georges Gomes FD, Marques Ramos AP, de Castro Jorge LA, Liesenberg V, et al. A CNN approach to simultaneously count plants and detect plantation-rows from UAV imagery. ISPRS J Photogramm Remote Sens. 2021;174:1–17.

[2] Huang Y, Qian Y, Wei H, Lu Y, Ling B, Qin Y. A survey of deep learning-based object detection methods in crop counting. Comput Electron Agric. 2023;215: Article 108425.

[3] Madec S, Jin X, Lu H, de Solan B, Liu S, Duyme F, Heritier E, Baret F. Ear density estimation from high resolution RGB imagery using deep learning technique. Agric For Meteorol. 2019;264:225–234.

[4] Bayraktar E, Basarkan ME, Celebi N. A low-cost UAV framework towards ornamental plant detection and counting in the wild. ISPRS J Photogramm Remote Sens. 2020;167:1–11.

[5] Du Y, Cai Y, Tan CW, Li Z, Yang G, Feng H, Dong H. Field wheat ears counting based on superpixel segmentation method. Sci Agric Sin. 2019;52:21–33.

[6] Liu T, Wu W, Chen W, Sun C, Zhu X, Guo W. Automated image-processing for counting seedlings in a wheat field. Precis Agric. 2016;17:392–406.

[7] Karami A, Quijano K, Crawford M. Advancing tassel detection and counting: Annotation and algorithms. Remote Sens. 2021;13(15):2881.

[8] Lu H, Cao Z, Xiao Y, Zhuang B, Shen C. TasselNet: Counting maize tassels in the wild via local counts regression network. Plant Methods. 2017;13:79. 29118821 10.1186/s13007-017-0224-0 PMC5664836

[9] Zheng H, Fan X, Bo W, Yang X, Tjahjadi T, Jin S. A multiscale point-supervised network for counting maize tassels in the wild. Plant Phenomics. 2023;5:0100. 37791249 10.34133/plantphenomics.0100 PMC10545326

[10] Kang R, Huang J, Zhou X, Ren N, Sun S. Toward real scenery: A lightweight tomato growth inspection algorithm for leaf disease detection and fruit counting. Plant Phenomics. 2024;6:0174. 38629080 10.34133/plantphenomics.0174 PMC11018486

[11] Li Y, Tang Y, Liu Y, Zheng D. Semi-supervised counting of grape berries in the field based on density mutual exclusion. Plant Phenomics. 2023;5:0115. 38033720 10.34133/plantphenomics.0115 PMC10684290

[12] Chen SW, Shivakumar SS, Dcunha S, das J, Okon E, Qu C, Taylor CJ, Kumar V. Counting apples and oranges with deep learning: A data-driven approach. IEEE Robot Autom Lett. 2017;2(2):781–788.

[13] Garcia-Garcia A, Orts-Escolano S, Oprea S, Villena-Martinez V, Martinez-Gonzalez P, Garcia-Rodriguez J. A survey on deep learning techniques for image and video semantic segmentation. Appl Soft Comput. 2018;70:41–65.

[14] Kestur R, Meduri A, Narasipura O. MangoNet: A deep semantic segmentation architecture for a method to detect and count mangoes in an open orchard. Eng Appl Artif Intell. 2019;77:59–69.

[15] Zabawa L, Kicherer A, Klingbeil L, Töpfer R, Kuhlmann H, Roscher R. Counting of grapevine berries in images via semantic segmentation using convolutional neural networks. ISPRS J Photogramm Remote Sens. 2020;164:73–83.

[16] Zou Z, Chen K, Shi Z, Guo Y, Ye J. Object detection in 20 years: A survey. Proc IEEE. 2023;111(3):257–276.

[17] Li H, Lee WS, Wang K. Immature green citrus fruit detection and counting based on fast normalized cross correlation (FNCC) using natural outdoor colour images. Precis Agric. 2016;17(6):678–697.

[18] Li Y, Ma R, Zhang R, Cheng Y, Dong C. A tea buds counting method based on YOLOV5 and Kalman filter tracking algorithm. Plant Phenomics. 2023;5:0030. 37011273 10.34133/plantphenomics.0030 PMC10062705

[19] Bai X, Liu P, Cao Z, Lu H, Xiong H, Yang A, Cai Z, Wang J, Yao J. Rice plant counting, locating, and sizing method based on high-throughput UAV RGB images. Plant Phenomics. 2023;5:0020. 37040495 10.34133/plantphenomics.0020 PMC10076056

[20] Zhang Y, Zhou D, Chen S, Gao S, Ma Y. Single-image crowd counting via multi-column convolutional neural network. Paper presented at: Proceedings of the IEEE Conference on Computer Vision and Pattern Recognition; 2016; Las Vegas, NV, USA.

[21] Fan Z, Zhang H, Zhang Z, Lu G, Zhang Y, Wang Y. A survey of crowd counting and density estimation based on convolutional neural network. Neurocomputing. 2022;472:224–251.

[22] Guo Y, Wu C, Du B, Zhang L. Density map-based vehicle counting in remote sensing images with limited resolution. ISPRS J Photogramm Remote Sens. 2022;189:201–217.

[23] Liao L, Xiao J, Yang Y, Ma X, Wang Z, Satoh S. High temporal frequency vehicle counting from low-resolution satellite images. ISPRS J Photogramm Remote Sens. 2023;198:45–59.

[24] Hao X, Cao Y, Zhang Z, Tomasetto F, Yan W, Xu C, Luan Q, Li Y. Countshoots: Automatic detection and counting of slash pine new shoots using UAV imagery. Plant Phenomics. 2023;5:0065. 38235123 10.34133/plantphenomics.0065 PMC10794053

[25] Lin J, Li J, Ma Z, Li C, Huang G, Lu H. A framework for single-panicle litchi flower counting by regression with multitask learning. Plant Phenomics. 2024;6:0172. 38629081 10.34133/plantphenomics.0172 PMC11018488

[26] Xiong H, Cao Z, Lu H, Madec S, Liu L, Shen C. TasselNetv2: In-field counting of wheat spikes with context-augmented local regression networks. Plant Methods. 2019;15:150. 31857821 10.1186/s13007-019-0537-2 PMC6905110

[27] Chen Y, Xin R, Jiang H, Liu Y, Zhang X, Yu J. Refined feature fusion for in-field high-density and multi-scale rice panicle counting in UAV images. Comput Electron Agric. 2023;211: Article 108032.

[28] Lin Z, Guo W. Sorghum panicle detection and counting using unmanned aerial system images and deep learning. Front Plant Sci. 2020;11: Article 534853. 32983210 10.3389/fpls.2020.534853 PMC7492560

[29] Oh Mh, Olsen P, Ramamurthy KN. Counting and segmenting sorghum heads. arXiv. 2019. 10.48550/arXiv.1905.13291.

[30] Ye J, Yu Z, Wang Y, Lu D, Zhou H. WheatLFANet: In-field detection and counting of wheat heads with high-real-time global regression network. Plant Methods. 2023;19(1):103. 37794515 10.1186/s13007-023-01079-x PMC10548667

[31] Zheng J, Fu H, Li W, Wu W, Zhao Y, Dong R, Yu L. Cross-regional oil palm tree counting and detection via a multi-level attention domain adaptation network. ISPRS J Photogramm Remote Sens. 2020;167:154–177.

[32] Liu L, Lu H, Li Y, Cao Z. High-throughput rice density estimation from transplantation to tillering stages using deep networks. Plant Phenomics. 2020;2020:1375957. 33313541 10.34133/2020/1375957 PMC7706318

[33] Ranjan V, Sharma U, Nguyen T, Hoai M. Learning to count everything. Paper presented at: Proceedings of the IEEE/CVF Conference on Computer Vision and Pattern Recognition; 2021; Nashville, TN, USA.

[34] You Z, Yang K, Luo W, Lu X, Cui L, Le X. Few-shot object counting with similarity-aware feature enhancement. Paper presented at: Proceedings of the IEEE/CVF Winter Conference on Applications of Computer Vision; 2023; Waikoloa, HI, USA.

[35] Shi M, Lu H, Feng C, Liu C, Cao Z. Represent, compare, and learn: A similarity-aware framework for class-agnostic counting. Paper presented at: Proceedings of the IEEE/CVF Conference on Computer Vision and Pattern Recognition; 2022; New Orleans, LA, USA.

[36] Ðukić N, Lukežič A, Zavrtanik V, Kristan M. A low-shot object counting network with iterative prototype adaptation. Paper presented at: Proceedings of the IEEE/CVF International Conference on Computer Vision; 2023; Paris, France.

[37] Ma J, Li Y, Liu H, Wu Y, Zhang L. Towards improved accuracy of UAV-based wheat ears counting: A transfer learning method of the ground-based fully convolutional network. Expert Syst Appl. 2022;191: Article 116226.

[38] Zhang T, Zhang X, Zhu P, Jia X, Tang X, Jiao L. Generalized few-shot object detection in remote sensing images. ISPRS J Photogramm Remote Sens. 2023;195:353–364.

[39] Qiu C, Zhang X, Tong X, Guan N, Yi X, Yang K, Zhu J, Yu A. Few-shot remote sensing image scene classification: Recent advances, new baselines, and future trends. ISPRS J Photogramm Remote Sens. 2024;209:368–382.

[40] Lu E, Xie W, Zisserman A. Class-agnostic counting. Paper presented at: Computer Vision–ACCV 2018: 14th Asian Conference on Computer Vision, Perth, Australia, December 2–6, 2018, Revised Selected Papers, Part III 14; 2019; Perth, Western Australia.

[41] Liu C, Zhong Y, Zisserman A, Xie W. Countr: Transformer-based generalised visual counting. arXiv. 2022. 10.48550/arXiv.2208.13721.

[42] Lin W, Yang K, Ma X, Gao J, Liu L, Liu S, Hou J, Yi S, Chan A. Scale-prior deformable convolution for exemplar-guided class agnostic counting. Paper presented at: BMVC; 2022; London, UK.

[43] Kirillov A, Mintun E, Ravi N, Mao H, Rolland C, Gustafson L, Xiao T, Whitehead S, Berg AC, Lo W-Y, et al. Segment anything. Paper presented at: Proceedings of the IEEE/CVF International Conference on Computer Vision; 2023; Paris, France.

[44] Dosovitskiy A, Beyer L, Kolesnikov A, Weissenborn D, Zhai X, Unterthiner T, Dehghani M, Minderer M, Heigold G, Gelly S, et al. An image is worth 16x16 words: Transformers for image recognition at scale. arXiv. 2020. 10.48550/arXiv.2010.11929.

[45] Ren S, He K, Girshick R, Sun J. Faster R-CNN: Towards real-time object detection with region proposal networks. IEEE Trans Pattern Anal Mach Intell. 2017;39(6):1137–1149. 27295650 10.1109/TPAMI.2016.2577031

[46] Woo S, Park J, Lee JY, Kweon IS. CBAM: Convolutional block attention module. Paper presented at: Proceedings of the European Conference on Computer Vision (ECCV); 2018; Munich, Germany.

[47] Yang SD, Su HT, Hsu WH, Chen WC. Class-agnostic few-shot object counting. Paper presented at: Proceedings of the IEEE/CVF Winter Conference on Applications of Computer Vision; 2021; Virtual.

[48] David E, Madec S, Sadeghi-Tehran P, Aasen H, Zheng B, Liu S, Kirchgessner N, Ishikawa G, Nagasawa K, Badhon MA, et al. Global wheat head detection (GWHD) dataset: A large and diverse dataset of high-resolution RGB-labelled images to develop and benchmark wheat head detection methods. Plant Phenomics. 2020;2020:3521852. 33313551 10.34133/2020/3521852 PMC7706323

[49] Lu H, Liu L, Li YN, Zhao XM, Wang XQ, Cao ZG. TasselNetV3: Explainable plant counting with guided upsampling and background suppression. IEEE Trans Geosci Remote Sens. 2021;60:1–15.

[50] Ghosal S, Zheng B, Chapman SC, Potgieter AB, Jordan DR, Wang X, Singh AK, Singh A, Hirafuji M, Ninomiya S, et al. A weakly supervised deep learning framework for sorghum head detection and counting. Plant Phenomics. 2019;2019:1525874. 33313521 10.34133/2019/1525874 PMC7706102

[51] Rashid MRA, Hossain MS, Fahim M, Islam MS, Tahzib-E-Alindo, Prito RH, Sheikh MSA, Ali MS, Hasan M, Islam M. Comprehensive dataset of annotated rice panicle image from Bangladesh. Data Brief. 2023;51: Article 109772. 38020434 10.1016/j.dib.2023.109772 PMC10661701

[52] Goldman E, Herzig R, Eisenschtat A, Goldberger J, Hassner T. Precise detection in densely packed scenes. Paper presented at: Proceedings of the IEEE/CVF Conference on Computer Vision and Pattern Recognition; 2019; Long Beach, CA, USA.

[53] Li Y, Zhang X, Chen D. Csrnet: Dilated convolutional neural networks for understanding the highly congested scenes. Paper presented at: Proceedings of the IEEE Conference on Computer Vision and Pattern Recognition; 2018; Salt Lake City, UT, USA.

[54] Jiang R, Liu L, Chen C. Clip-count: Towards text-guided zero-shot object counting. Paper presented at: Proceedings of the 31st ACM International Conference on Multimedia; 2023; Ottawa, Canada.

[55] Deng J, Dong W, Socher R, Li LJ, Li K, Fei-Fei L. Imagenet: A large-scale hierarchical image database. Paper presented at: 2009 IEEE Conference on Computer Vision and Pattern Recognition; 2009; Miami, FL, USA.

[56] Wu J, Yang G, Yang X, Xu B, Han L, Zhu Y. Automatic counting of in situ rice seedlings from UAV images based on a deep fully convolutional neural network. Remote Sens. 2019;11(6):691.

